# HTLV-1 Tax Stabilizes MCL-1 via TRAF6-Dependent K63-Linked Polyubiquitination to Promote Cell Survival and Transformation

**DOI:** 10.1371/journal.ppat.1004458

**Published:** 2014-10-23

**Authors:** Young Bong Choi, Edward William Harhaj

**Affiliations:** Department of Oncology, Sidney Kimmel Comprehensive Cancer Center, Johns Hopkins School of Medicine, Baltimore, Maryland, United States of America; University of Pennsylvania School of Medicine, United States of America

## Abstract

The human T-cell leukemia virus type 1 (HTLV-1) Tax protein hijacks the host ubiquitin machinery to activate IκB kinases (IKKs) and NF-κB and promote cell survival; however, the key ubiquitinated factors downstream of Tax involved in cell transformation are unknown. Using mass spectrometry, we undertook an unbiased proteome-wide quantitative survey of cellular proteins modified by ubiquitin in the presence of Tax or a Tax mutant impaired in IKK activation. Tax induced the ubiquitination of 22 cellular proteins, including the anti-apoptotic BCL-2 family member MCL-1, in an IKK-dependent manner. Tax was found to promote the nondegradative lysine 63 (K63)-linked polyubiquitination of MCL-1 that was dependent on the E3 ubiquitin ligase TRAF6 and the IKK complex. Tax interacted with and activated TRAF6, and triggered its mitochondrial localization, where it conjugated four carboxyl-terminal lysine residues of MCL-1 with K63-linked polyubiquitin chains, which stabilized and protected MCL-1 from genotoxic stress-induced degradation. TRAF6 and MCL-1 played essential roles in the survival of HTLV-1 transformed cells and the immortalization of primary T cells by HTLV-1. Therefore, K63-linked polyubiquitination represents a novel regulatory mechanism controlling MCL-1 stability that has been usurped by a viral oncogene to precipitate cell survival and transformation.

## Author Summary

HTLV-1 infection is etiologically linked to the development of the neuroinflammatory disorder HTLV-1 associated myelopathy/tropical spastic paraparesis (HAM/TSP) and adult T-cell leukemia (ATL), an aggressive CD4+CD25+ malignancy. The HTLV-1 regulatory protein Tax constitutively activates the IκB kinases (IKKs) and NF-κB to promote cell survival, proliferation and transformation. However, the precise mechanisms by which Tax and IKK regulate cell survival are largely unknown. Here, we found that Tax interacts with and activates the host ubiquitin ligase TRAF6, and promotes a redistribution of TRAF6 to the mitochondria. TRAF6 conjugates the anti-apoptotic BCL-2 family member MCL-1 with lysine 63 (K63)-linked polyubiquitin chains that antagonize MCL-1 interaction with the 20S proteasome, thereby protecting MCL-1 from degradation elicited by chemotherapeutic drugs. TRAF6 and MCL-1 both played pivotal roles in the survival of ATL cells and the immortalization of primary T cells by HTLV-1. Overall, our study has identified a novel TRAF6/MCL-1 axis that has been subverted by the HTLV-1 Tax protein to maintain the survival of HTLV-1 infected T cells.

## Introduction

Human T-cell leukemia virus 1 (HTLV-1) infects approximately 20 million people worldwide and is the etiological agent of adult T-cell leukemia (ATL), an aggressive CD4+CD25+ malignancy that occurs in a small percentage of infected individuals after a long latent period [1]. HTLV-1 infection is also associated with a host of inflammatory diseases including HTLV-1-associated myelopathy/tropical spastic paraparesis (HAM/TSP). HTLV-1 Tax is a key regulatory protein essential for viral gene expression by recruiting CREB/ATF transcription factors to the viral long terminal repeats (LTRs) [2]. Tax also plays a central role in cell transformation by HTLV-1 and is sufficient to immortalize primary human T lymphocytes [3]. Furthermore, transgenic mice expressing Tax in the T-cell compartment develop leukemia and lymphoma with clinical and pathological features resembling ATL [4]. Tax stimulates the proliferation, survival and immortalization of T cells by inactivating tumor suppressors, promoting cell cycle progression and activating anti-apoptotic pathways [5]. One of the principal cellular pathways targeted by Tax and essential for Tax-mediated transformation is NF-κB [6].

NF-κB is composed of heterodimeric DNA binding proteins containing RelA, c-Rel, RelB, p50 and p52 [7]. NF-κB is held inactive in the cytoplasm by members of the IκB family, all of which contain ankyrin repeat domains. In the canonical NF-κB pathway, a wide variety of stimuli including proinflammatory cytokines and stress signals converge on the IκB kinase (IKK) complex consisting of the catalytic subunits IKKα and IKKβ and the regulatory subunit IKKγ (also known as NEMO) [8]. IKKβ phosphorylates the NF-κB inhibitor IκBα to trigger its ubiquitination and degradation by the proteasome thus allowing NF-κB to translocate to the nucleus and activate anti-apoptotic and pro-inflammatory target genes [9]. In the noncanonical NF-κB pathway, specific tumor necrosis factor receptor (TNFR) superfamily members including BAFF, lymphotoxin-β and CD40 induce the proteasome-dependent processing of the p100 (NF-κB2) precursor to yield p52, which heterodimerizes with RelB to activate a distinct gene program [10]. Tax constitutively activates both canonical and noncanonical NF-κB pathways, in part by interacting with NEMO [11], [12]. HTLV-1 molecular clones bearing Tax mutants defective for NF-κB activation are impaired in T-cell immortalization [6]. HTLV-1 transformed cell lines and primary ATL cells all exhibit constitutive NF-κB activation that is integral for the survival of these virally transformed lymphocytes [13]. Since Tax expressing cells are vigorously targeted for elimination by cytotoxic T cells, the majority (∼60%) of ATL tumors exhibit downregulated or lost Tax expression, either by mutations within Tax or deletion or methylation of the 5′ LTR [14].

NF-κB activation is tightly regulated by post-translational modifications, with ubiquitin playing a prominent role in both canonical and noncanonical NF-κB pathways. Ubiquitin (Ub) is conjugated to a lysine residue in a substrate by the sequential process of three enzymes: Ub-activating enzyme (E1), Ub-conjugating enzyme (E2), and Ub ligase (E3) [15]. Ubiquitin contains seven lysine residues (K6, 11, 27, 29, 33, 48, 63), each of which can support the elongation of polyubiquitin (polyUb) chains [16]. K48-linked polyUb chains direct substrates to the proteasome for degradation, whereas K63-linked polyUb chains mainly serve nondegradative roles including receptor trafficking, DNA damage repair, kinase activation and signal transduction [17]. In the NF-κB pathway, K63-linked polyUb chains conjugated to adaptor proteins (e.g. RIP1) provide platforms for the recruitment of TAK1 (TGF-β activating kinase 1) and IKK kinase complexes that signal downstream NF-κB activation [18]. Dysregulation of the ubiquitination machinery, in particular the E3 ligases that direct a protein to proteasomal degradation, may lead to uncontrolled cell growth and tumorigenesis [19].

HTLV-1 Tax is conjugated with K63-linked polyUb chains which plays an essential role in NEMO binding and Tax activation of IKK and NF-κB [20], [21]. The E2 enzyme Ubc13 is required for Tax ubiquitination [20], however the E3 enzyme that ubiquitinates Tax has yet to be identified. Although Tax hijacks ubiquitin for NF-κB activation, whether Tax utilizes the ubiquitin machinery for distinct events in the transformation process remains unknown. We postulated that ubiquitination events downstream of Tax might play important roles in HTLV-1-induced cellular transformation; therefore, a proteome-wide quantitative survey of cellular proteins modified by ubiquitin in the presence of Tax was undertaken. This endeavor led to the identification of the anti-apoptotic myeloid cell leukemia-1 (MCL-1) protein as a key target that was ubiquitinated in a TRAF6-dependent manner downstream of Tax. Tax interacted with TRAF6 and facilitated its mitochondrial localization where it conjugated MCL-1 with K63-linked polyubiquitination chains to enhance MCL-1 stability and protection from genotoxic stress-induced degradation.

## Results

### Tax promotes the K63-linked polyubiquitination of MCL-1

**Figure 1:**
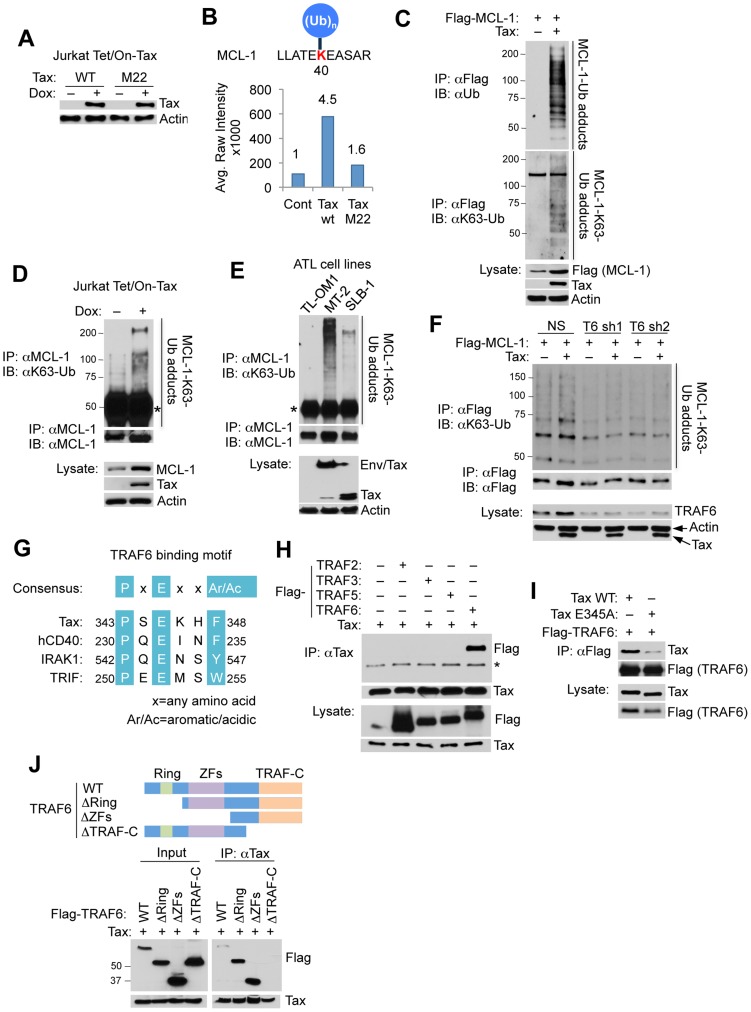
Tax promotes TRAF6-dependent MCL-1 ubiquitination. (A) Doxycycline (Dox)-induced expression of Tax wild-type (WT) and M22 in Jurkat Tet/On-Tax cells as shown by immunoblotting. (B) UbiScan analysis of Tax-induced MCL-1 ubiquitination at lysine 40 (K40). The relative intensity of peak (651.85 m/z) is marked on the top of each bar. (C–F) Tax induces the K63-linked polyubiquitination of MCL-1. Ubiquitination assay was performed with immunoprecipitates (IPs) derived from 293T cells transfected with Flag-MCL-1 with or without Tax (C), Jurkat Tet/On-Tax cells treated with Dox for 48 h (D), the indicated HTLV-1 transformed cell lines (E), and 293T cells transfected with Flag-MCL-1 together with shRNA (NS), TRAF6 shRNA 1 (T6 sh1), or TRAF6 shRNA 2 (T6 sh2) with or without Tax (F). “Env/Tax” indicates the Env-Tax fusion protein expressed in MT-2 cells (E). Asterisk (*) indicates immunoglobulin heavy chain (D and E). (G) Schematic of the TRAF6 binding motif of Tax. Highlighted in turquoise are consensus sequences among the indicated proteins. (H) Specific interaction of MCL-1 and TRAF6. Co-IP analysis was performed with Tax immunoprecipitates from lysates of 293T cells transfected with Tax together with Flag-TRAF2, TRAF3, TRAF5 or TRAF6. * indicates IgG heavy chain. (I) Mapping of the TRAF6 binding domain in Tax. Co-IP analysis was performed with Flag immunoprecipitates from lysates of 293T cells transfected with Flag-TRAF6 together with Tax WT or E345A. (J) Mapping of the Tax binding domain in TRAF6. Co-IP analysis was performed with Tax immunoprecipitates from lysates of 293T cells transfected with Tax together with Flag-TRAF6 WT, ΔRING (275–522 residues) lacking the RING finger domain, ΔZFs (113–522 residues) lacking zinc finger (ZF) domains, or ΔTRAF-C (1–351 residues) lacking the TRAF-C domain as shown. Western blot was performed with rabbit anti-Flag antibody.

To identify cellular proteins ubiquitinated in a Tax-dependent manner Jurkat cells expressing tetracycline-inducible Tax and Tax^M22^, a mutant (T130A, L131S) impaired in NEMO binding and NF-κB activation [11], [22], were subjected to the UbiScan ubiquitination proteomics platform. Ubiquitinated peptides were enriched from cell lysates using the ubiquitin branch (“K-ε-GG”) antibody and subjected to liquid chromatography-tandem mass spectrometry (LC-MS/MS) analysis. Tax expression was confirmed by immunoblotting in Tax and Tax^M22^-induced cells (Figure 1A). A total of 136 proteins (total of 204 Ub sites) were identified with ubiquitination profiles modulated in response to Tax expression (Table S1). Furthermore, 22 of these candidates, including the anti-apoptotic BCL-2 family member MCL-1, were ubiquitinated in a Tax and IKK-dependent manner (by Tax but not Tax^M22^)(Figure 1B). MCL-1 is localized in the mitochondria and directly binds and antagonizes pro-apoptotic BCL-2 family members BAX and BAK, as well as BH3 only family members BIM, BID, NOXA and PUMA, to restrain cytochrome C release from the mitochondria and inhibit apoptosis [23].

MCL-1 has an amino (N)-terminal extended PEST (rich in proline (P), glutamic acid (E), serine (S) and threonine residues (T)) domain that regulates its stability [24]. MCL-1 is a highly labile protein with a half-life of 2–4 hours, and its expression is rapidly diminished in response to apoptotic stimuli, mainly through the ubiquitin-proteasome degradation pathway [25]–[29]. To determine the type of polyUb chains conjugated onto MCL-1 in response to Tax expression, we performed an ubiquitination assay with HA-tagged wild-type (WT), K48-only, or K63-only Ub plasmids. MCL-1 was conjugated predominantly with K63-linked polyUb, and with a lesser extent K48-linked polyUb when co-expressed with Tax (Figure S1). This result was confirmed with endogenous ubiquitin chains by immunoblotting with a K63-Ub linkage-specific antibody in Tax-transfected 293T cells, Tax-inducible Jurkat Tet/On-Tax cells, and the HTLV-1 transformed T-cell lines MT-2, SLB-1 and TL-OM1 (Figures 1C–E). TL-OM1 is an ATL cell line lacking Tax expression [13]. MT-2 and SLB-1 cells express Tax; in addition MT-2 cells also harbor an envelope (Env)/Tax fusion (Figure 1E). MCL-1 K63-linked polyubiquitination correlated with Tax expression, with MT-2 cells exhibiting higher levels of MCL-1 ubiquitination compared to SLB-1 and TL-OM1 (Figure 1E). To further confirm that the K63-linked polyubiquitination observed was specific to MCL-1, and not an artifactual result caused by an associated protein, a ubiquitination assay was conducted with transfected His-tagged MCL-1. His-MCL-1 was precipitated from lysates with Ni-NTA agarose beads and washed with buffer containing 8 M urea to eliminate any MCL-1 associated proteins. Consistent with earlier results, Tax specifically induced the K63-linked polyubiquitination of His-MCL-1 (Figure S2).

Among K63-Ub specific E3 ligases, TRAF6 autoubiquitination appears to be enhanced by Tax expression [30]. Consistent with this report, our *in vitro* ubiquitination assays revealed that TRAF6 was ubiquitinated when incubated with purified Tax protein (Figure S3). However, TRAF6^C70A^, a RING domain point mutant impaired in E3 ligase activity, was not ubiquitinated in response to Tax (Figure S3), indicating that Tax triggers the enzymatic activity and autoubiquitination of TRAF6. Moreover, Tax lost its ability to induce MCL-1 K63-linked polyubiquitination in cells expressing two distinct TRAF6 short hairpin RNAs (shRNAs) (Figure 1F), indicating that Tax activation of TRAF6 is critical for MCL-1 ubiquitination. By inspection of the Tax protein sequence, we identified *in silico* a putative TRAF6 binding motif [31] in the carboxyl (C)-terminal tail of Tax (Figure 1G). Interestingly, the putative TRAF6 binding motif lies within a domain essential for Tax transformation and is immediately adjacent to the PDZ binding motif (PBM) of Tax [32]. Indeed, co-immunoprecipitation (co-IP) analysis revealed that Tax specifically interacted with TRAF6 and not other TRAF proteins, but the interaction with TRAF6 was substantially diminished when the conserved glutamic acid residue in the TRAF6 binding motif was substituted with alanine (Tax^E345A^) (Figure 1H and I). Conversely, TRAF6 did not interact with Tax when the TRAF-C domain of TRAF6, a region that regulates oligomerization of TRAF6 and interaction with upstream signaling molecules, was deleted (Figure 1J). Together, these results indicate that Tax induces the K63-linked polyubiquitination of MCL-1 and interacts with TRAF6 via a specific C-terminal motif.

### Tax activates TRAF6 and promotes its mitochondrial localization

**Figure 2:**
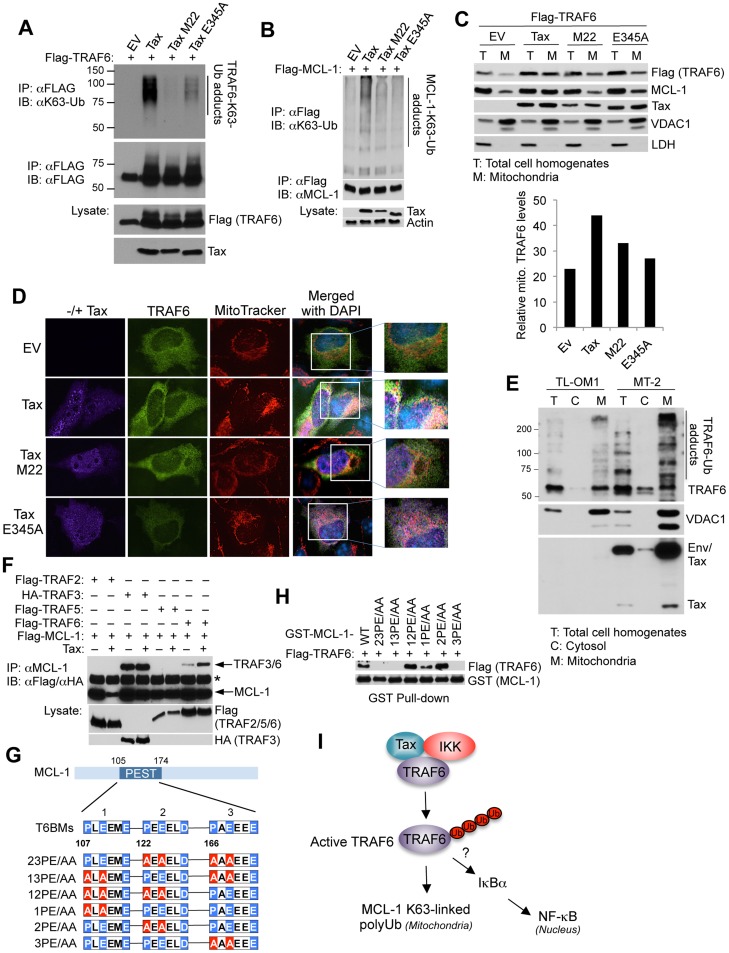
Tax activates TRAF6 and enhances its interaction with MCL-1 in the mitochondria. (A) Tax requires NEMO and TRAF6 binding to activate TRAF6. Ubiquitination assay was performed with Flag immunoprecipitates from lysates of 293T cells transfected with Flag-TRAF6 along with empty vector (EV), Tax and Tax point mutants M22 and E345A. (B) Tax requires NEMO and TRAF6 binding to promote MCL-1 K63-linked polyubiquitination. Ubiquitination assay was performed with Flag immunoprecipitates from lysates of 293T cells transfected with Flag-MCL-1 together with Tax WT, M22 and E345A. (C, D) Tax induces the mitochondrial localization of TRAF6. (C) Immunoblotting was performed with the indicated fractions derived from 293 cells transfected with Flag-TRAF6 together with Tax WT, M22 or E345A. Fifty-fold excess of mitochondrial extracts compared to total cell homogenates were loaded onto the gel for normalization. Voltage-dependent anion channel 1 (VDAC1) and lactate dehydrogenase (LDH) were used as markers for mitochondria and cytosol, respectively. Mitochondrial TRAF6 was quantitated using Alpha Innotech gel imaging software. Relative intensity of mitochondrial TRAF6 bands normalized to VDAC1 was calculated by dividing by the normalized band intensity of total TRAF6. (D) Immunofluorescence assay was performed with HeLa cells transfected with Flag-TRAF6 along with Tax WT, M22 or E345A and incubated with MitoTracker Red for 30 min prior to fixation. Nuclei were counterstained with DAPI (blue) before mounting coverslips. (E) Endogenous TRAF6 is ubiquitinated and localized in the mitochondria in HTLV-1 transformed cells. Immunoblotting was performed with the indicated fractions derived from TL-OM1 and MT-2 cells. Fifty fold excess of mitochondrial extracts compared to total cell homogenates and cytosol were loaded onto the gel for normalization. VDAC1 and LDH served as markers for mitochondria and cytosol, respectively. (F) Tax induces TRAF6 and MCL-1 interactions. Immunoblotting was performed with MCL-1 immunoprecipitates from lysates of 293T cells transfected with Flag-MCL-1 together with Flag-TRAF2, TRAF5 or TRAF6, or HA-TRAF3, with or without Tax. Asterisk (*) indicates immunoglobulin heavy chain. (G) Three identified TRAF6 binding motifs (T6BMs) in the MCL-1 PEST domain. Consensus sequences of the T6BMs in blue were substituted for alanine in red as shown in the diagram. (H) Mapping of the T6BMs of MCL-1. GST pull-down assay was performed using purified Flag-TRAF6 and GST-MCL-1 WT or mutants as indicated. (I) Proposed model of Tax, IKK and TRAF6 regulation of MCL-1 K63-linked polyubiquitination.

We next examined the effect of Tax mutants impaired in IKK activation (M22) or TRAF6 binding (E345A) in the activation of TRAF6 by conducting TRAF6 auto-ubiquitination assays. Tax promoted the K63-linked autoubiquitination of TRAF6, however Tax^M22^ and Tax^E345A^ mutants were both impaired in inducing the TRAF6 autoubiquitination (Figure 2A). Tax^E345A^ only weakly activated TRAF6, likely due to the residual binding of this mutant with TRAF6 (Figure 1I). These results indicate that Tax requires both IKK/NEMO and TRAF6 binding to activate TRAF6. Furthermore, both Tax^M22^ and Tax^E345A^ were deficient in promoting MCL-1 K63-linked polyubiquitination (Figure 2B). Therefore, Tax also requires interactions with IKK/NEMO and TRAF6 to induce the K63-linked polyubiquitination of MCL-1. TRAF6 likely directly ubiquitinates MCL-1 since purified TRAF6 promoted the ubiquitination of recombinant GST-MCL-1 and co-expression of Tax with TRAF6 further enhanced MCL-1 ubiquitination (Figure S4).

To further understand how Tax interaction with TRAF6 regulated MCL-1 ubiquitination, we examined the subcellular localization of TRAF6 since it has been shown to traffic to the mitochondria as part of its regulation of host innate immune signaling or mitochondria quality control [33], [34]. Indeed, Tax specifically induced the mitochondrial translocation of TRAF6 but not other TRAFs (Figure S5). We next conducted biochemical fractionation and immunofluorescence assays to examine the effect of Tax mutants on TRAF6 translocation to the mitochondria. Tax^M22^ and Tax^E345A^ were less effective than wild-type Tax in facilitating the translocation of TRAF6 to mitochondria (Figure 2C and D). Tax^M22^ and Tax^E345A^ were also deficient in the stabilization of MCL-1 (Figure 2C). Interestingly, a significant fraction of Tax was found in the mitochondria where it partially co-localized with TRAF6 (Figure 2C and D). However, TRAF6^ΔTRAF-C^, which does not interact with Tax (Figure 1J), did not translocate to the mitochondria in response to Tax expression (Figure S6). TRAF6 was mostly localized in the mitochondria in HTLV-1 transformed MT-2 cells and was heavily modified, most likely by polyUb chains (Figure 2E). The Tax- ATL cell line TL-OM1 exhibited less pronounced TRAF6 modification compared to MT-2 cells (Figure 2E). Based on these collective findings, we postulated that Tax could mediate the interaction between TRAF6 and MCL-1. Indeed, co-IP analysis revealed that Tax specifically enhanced the interaction between MCL-1 and TRAF6 (Figure 2F). Although MCL-1 interacted with TRAF3 under basal conditions, Tax had no effect on this interaction (Figure 2F). Three conserved TRAF6 binding motifs within the PEST domain of MCL-1 were identified and mutations were rendered within each motif as indicated, both singly and in combination (Figure 2G). Notably, the other BCL-2 family members BCL-2, BCL-x(L) and BFL-1/A1 all lack TRAF6 binding sites. *In vitro* GST pull-down assays revealed that the third TRAF6 binding motif was most critical for MCL-1 binding to TRAF6 since all mutants harboring mutations in the third TRAF6 binding motif failed to interact with TRAF6 (Figure 2H). Taken together, Tax forms a complex with IKK and TRAF6 and triggers TRAF6 redistribution to the mitochondria where it conjugates MCL-1 with K63-linked polyUb chains (Figure 2I). The collective data also raise the possibility that TRAF6 may play a role in Tax-mediated NF-κB activation.

### Tax stabilizes MCL-1 through an IKK and TRAF6-dependent mechanism

**Figure 3:**
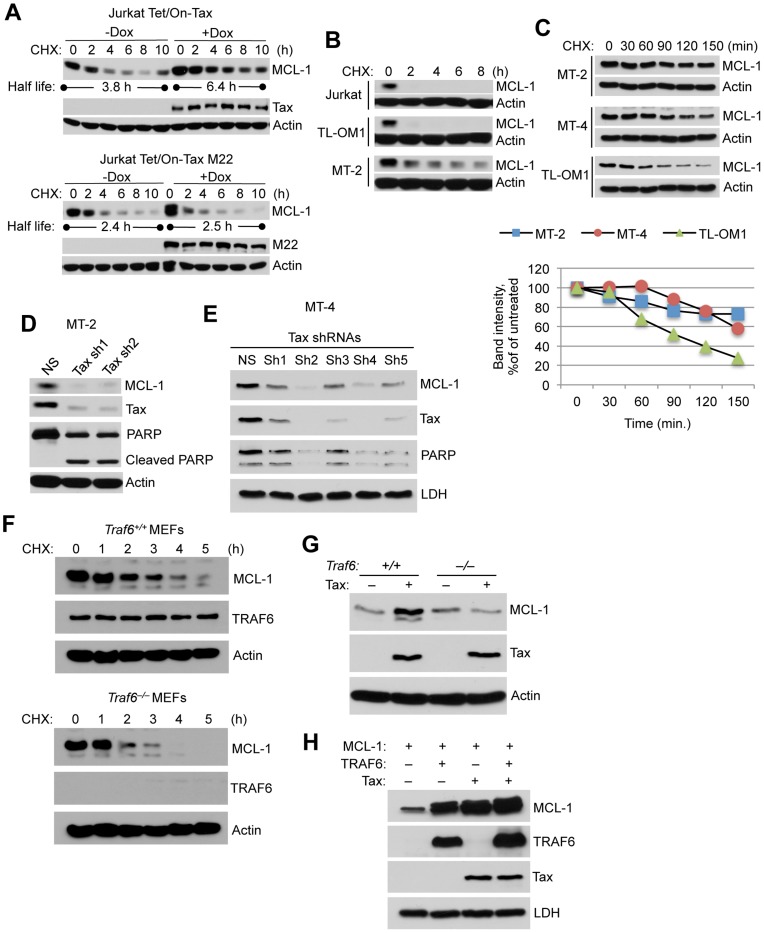
Tax stabilizes MCL-1 in an IKK and TRAF6-dependent manner. (A) Tax prolongs the half-life of MCL-1. CHX chase assay was performed with Jurkat Tet/On-Tax or M22 cells cultured in the presence or absence of Dox for 48 h and subsequently treated with CHX (100 µg/ml) for the indicated times. The half-life of MCL-1 was calculated from a linear least-square fit of the protein intensity. (B, C) MCL-1 is stabilized in Tax-expressing cell lines. CHX chase assays were performed by immunoblotting with whole cell lysates derived from Jurkat and HTLV-1 transformed cell lines including TL-OM1, MT-2 and MT-4 at the indicated times after CHX treatment (10 µg/ml). Band intensity was measured using Alpha Innotech gel imaging software. The relative intensity of MCL-1 bands was calculated compared to actin. (D, E) Depletion of Tax with shRNAs results in the loss of MCL-1 and apoptosis in HTLV-1 transformed cells. Immunoblotting was performed with the indicated antibodies using whole cell lysates derived from MT-2 (D) and MT-4 (E) cells cultured for 6 days after the lentiviral transduction of Tax shRNAs. (F) TRAF6 is essential for MCL-1 stabilization. CHX chase assay was performed by immunoblotting with whole cell lysates derived from *Traf6^+/+^* and *Traf6^−/−^* MEFs at the indicated times after CHX treatment. (G) Tax requires TRAF6 for MCL-1 stabilization. Immunoblotting was performed using lysates from *Traf6^+/+^* and *Traf6^−/−^* MEFs transfected with Tax. (H) Increased stabilization of MCL-1 by Tax and TRAF6. Immunoblotting was performed using lysates from 293 cells transfected with MCL-1, TRAF6 and Tax.

MCL-1 is a highly labile protein due to phosphodegron motifs in the PEST domain targeted by the FBW7 (F-box and WD repeat domain-containing 7) E3 ligase complex [28], [29]. Cycloheximide (CHX) chase assays were next conducted to examine MCL-1 stability, which revealed that Tax, but not Tax^M22^, significantly prolonged the half-life of MCL-1 in Jurkat cells (Figure 3A). Consistent with these results, Tax failed to enhance the half-life of MCL-1 protein in NEMO-deficient Jurkat cells (Figure S7), indicating that Tax requires IKK to stabilize MCL-1. MCL-1 was more stable in Tax+ MT-2 cells compared to the Tax- ATL cell line TL-OM-1 and Jurkat cells (Figure 3B). MCL-1 stability was also increased in Tax-expressing MT-4 cells compared to TL-OM1 cells (Figure 3C). Tax depletion by two distinct shRNAs triggered the loss of MCL-1 in MT-2 cells together with cleavage of poly ADP ribose polymerase (PARP), indicative of apoptosis (Figure 3D). Consistent with these results, shRNA-mediated knockdown of Tax in MT-4 cells with shRNAs #2 and 4 correlated with loss of MCL-1 and PARP (Figure 3E). Therefore, MCL-1 protein levels are under the strict control of Tax in HTLV-1 transformed cell lines, and furthermore Tax is dependent on IKK to stabilize MCL-1.

We next examined the stability of MCL-1 by CHX chase assays in murine embryonic fibroblasts (MEFs) with a genetic deletion of TRAF6 [35]. MCL-1 stability was sharply diminished in *Traf6* ^−/−^ MEFs compared to control wild-type MEFs (Figure 3F). Although Tax stabilized endogenous MCL-1 in wild-type MEFs, Tax had no effect on MCL-1 stability in *Traf6* ^−/−^ MEFs (Figure 3G). We next examined if Tax and TRAF6 acted synergistically to stabilize MCL-1. Overexpression of either Tax or TRAF6 stabilized MCL-1 in 293 cells as expected, and Tax and TRAF6 together further increased MCL-1 stability (Figure 3H). Taken together, our data provides strong evidence that TRAF6 plays a central role in regulating the stability of MCL-1.

### Tax protects MCL-1 from proteasomal degradation induced by genotoxic stress stimuli

**Figure 4:**
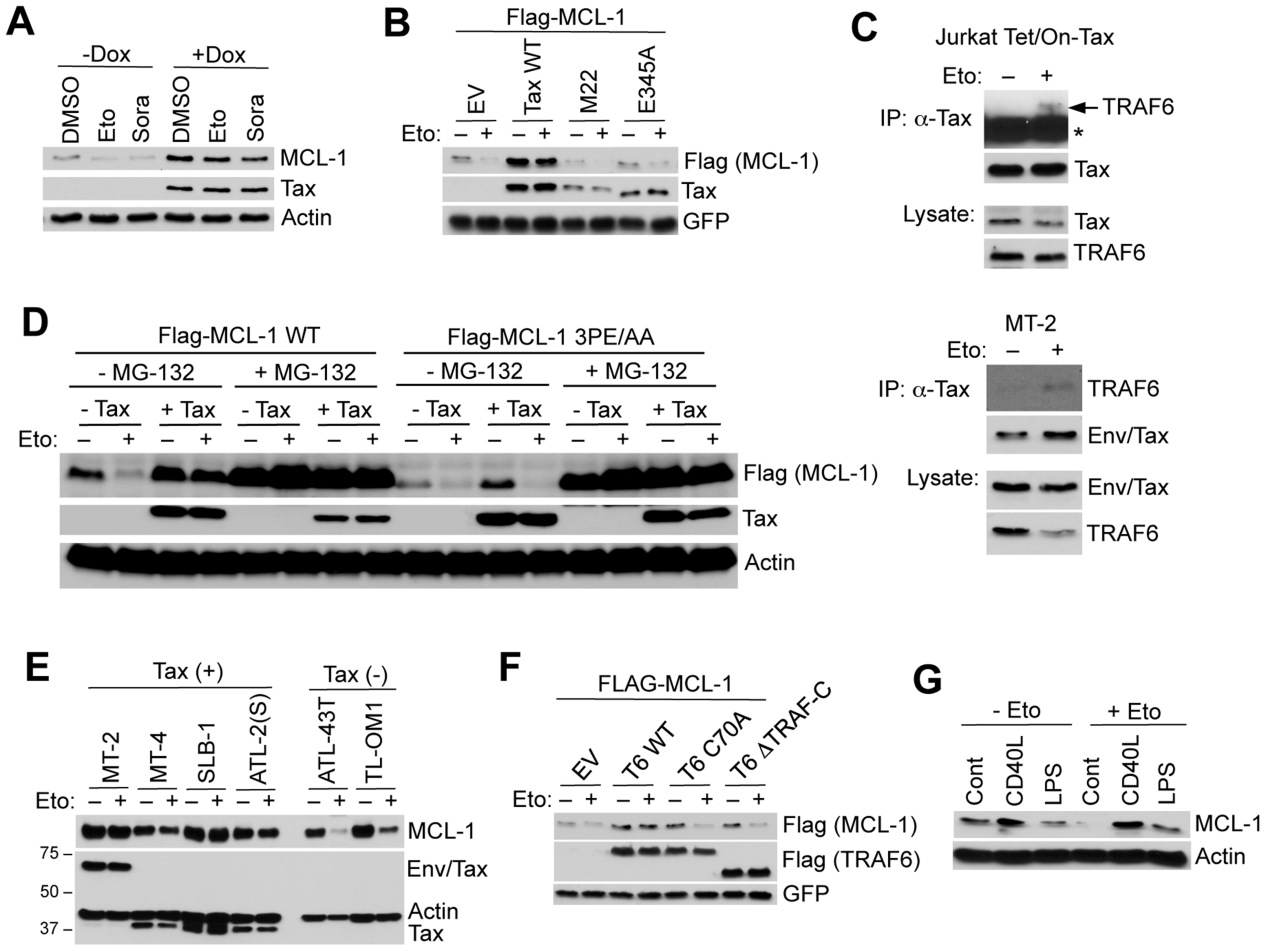
Tax and TRAF6 protect MCL-1 from degradation induced by genotoxic stress stimuli. (A) Tax protects MCL-1 from genotoxic stress-induced degradation. Immunoblotting was performed with whole cell lysates derived from Jurkat Tet/On-Tax cells cultured in the presence or absence of Dox for 48 h and subsequently treated with etoposide (Eto, 10 µg/ml) or sorafenib (Sora, 10 µM) for 12 h. (B) 293 cells transfected with Flag-MCL-1 and GFP together with Tax WT, M22 or E345A were treated with etoposide for 24 h. (C) Etoposide treatment enhances the interactions between endogenous TRAF6 and Tax proteins. TRAF6 immunoblotting was performed with Tax immunoprecipitates from lysates of Jurkat Tet/On-Tax cells incubated with Dox for 48 h (left panel) and MT-2 cells (right panel). The cells were treated with etoposide (10 µM) for 6 h before harvesting. Asterisk (*) indicates immunoglobulin heavy chain. (D) Tax-induced MCL-1 stabilization and protection is dependent on TRAF6-binding motif #3 of MCL-1. Immunoblotting was performed with whole cell lysates derived from 293 cells transfected with Flag-MCL-1 WT or 3PE/AA mutant together with empty vector or Tax and left untreated or treated with MG-132 (10 µM) for 24 h before harvesting. (E) Tax+ cell lines are protected from etoposide-induced MCL-1 degradation. HTLV-1 transformed and ATL cell lines (Tax+ or Tax-) cultured in the presence or absence of etoposide for 24 h. Immunoblotting was performed with the indicated antibodies. (F) TRAF6 protects MCL-1 from etoposide-induced degradation. Immunoblotting was performed with whole cell lysates derived from 293 cells transfected with Flag-MCL-1 and GFP together with Flag-TRAF6 WT, C70A or ΔTRAF-C, and then treated with etoposide for 24 h. (G) CD40L and LPS protect MCL-1 from etoposide-induced degradation in primary B cells. Immunoblotting was performed with whole cell lysates derived from primary murine splenic B cells cultured in the presence of CD40L or LPS for 24 h, and then treated with etoposide for an additional 24 h.

We next examined if Tax was able to protect MCL-1 from degradation in response to stimuli that trigger genotoxic stress and apoptosis. Indeed, Tax prevented MCL-1 degradation induced by ultraviolet irradiation and DNA-damaging drugs including the topoisomerase II inhibitor etoposide, and the kinase inhibitor sorafenib (Figures 4A and S8). However, Tax^M22^ and Tax^E345A^ mutants failed to prevent etoposide-induced degradation of MCL-1 (Figure 4B). Interestingly, etoposide treatment enhanced the interactions between endogenous TRAF6 and Tax proteins in Tax inducible Jurkat Tet/On-Tax and MT-2 cells as shown by co-IP (Figure 4C). The third TRAF6 binding motif in MCL-1, that we previously demonstrated mediated the interaction with TRAF6 (Figure 2H), was essential for Tax to prevent etoposide-induced MCL-1 degradation (Figure 4D). Furthermore, shRNA-mediated depletion of IKKα and IKKβ or inhibition of IKKβ with SC-514, a small molecule IKKβ inhibitor, restored the sensitivity of MT-2 cells to etoposide-induced degradation of MCL-1 (Figure S9A–C). These results provide further evidence that IKK serves a critical role in the protection of MCL-1 from degradation triggered by genotoxic stress. Biochemical fractionation studies using Tax inducible Jurkat cells revealed that Tax increased the mitochondrial localization of NEMO, IKKβ and TRAF6 (Figure S9D), thus raising the possibility that IKK may regulate TRAF6 and/or MCL-1 in the mitochondria.

We next examined if Tax induced the mRNA expression of MCL-1 by quantitative real-time PCR (qRT-PCR). MCL-1 was not transcriptionally activated by Tax, although cIAP2 and BFL-1 were strongly induced by Tax as previously described [36], [37] (Figure S10). Tax+ HTLV-1 transformed cell lines also did not exhibit elevated levels of MCL-1 mRNA compared to Jurkat or the Tax- ATL cell line ED40515(-) (Figure S11). Together these results strongly suggest that Tax regulates MCL-1 chiefly by post-translational mechanisms.

Etoposide is currently a component of the regimen of conventional chemotherapy for ATL patients, nevertheless acute ATL carries a dismal prognosis due to rapid emergence of chemotherapy resistance [38]. Our data revealed that Tax+ HTLV-1 transformed cell lines (MT-2, MT-4, SLB-1 and ATL-2(S)), but not Tax- ATL cells (ATL-43T and TL-OM1), were highly refractory to etoposide-induced MCL-1 degradation (Figure 4E). Thus, Tax expression may contribute to chemotherapy drug resistance in ATL. It was next examined if TRAF6 required intact RING and TRAF domains to protect MCL-1 from stress-induced degradation. Overexpression of wild-type TRAF6, but not the RING mutant TRAF6^C70A^ or TRAF deletion TRAF6^ΔTRAF-C^, stabilized and protected MCL-1 from etoposide-induced degradation (Figure 4F). Thus, TRAF6 requires its E3 activity and oligomerization domain to mitigate the effects of genotoxic stress on MCL-1 stability.

Given that TRAF6 functions as an essential signaling molecule downstream of Toll-like receptors (TLRs) and the costimulatory molecule CD40, it is plausible that Tax may have hijacked a TRAF6-dependent signaling pathway that controls MCL-1 stability during immune activation. Thus, we examined if TRAF6 activating stimuli including CD40 ligand (CD40L) and lipopolysaccharide (LPS) influenced MCL-1 stability. Indeed, CD40L and LPS pre-treatment prevented etoposide-induced MCL-1 degradation in primary mouse B cells in the absence of transcriptional induction of MCL-1 (Figures 4G and S12). However, CD40L and LPS treatment strongly induced ICAM-1 and A20 mRNAs (Figure S12). The Epstein-Barr virus (EBV)-encoded latent membrane protein 1 (LMP1) is a potent activator of TRAF6 and also protected MCL-1 from etoposide-induced degradation (Figure S13). Moreover, LPS induced the K63-linked polyubiquitination of MCL-1 in a TRAF6-dependent manner in RAW 264.7 mouse macrophages (Figure S14). Thus, TRAF6 stabilization of MCL-1 may represent a common mechanism of cell survival upon activation of innate immune signaling pathways.

### TRAF6 conjugates C-terminal lysines in MCL-1 with K63-linked polyUb chains

**Figure 5:**
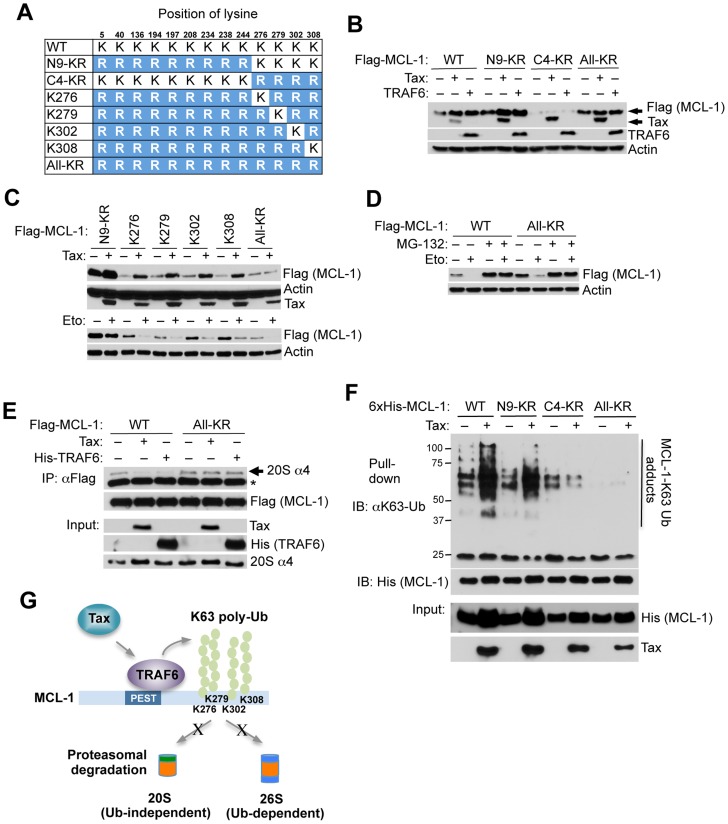
Tax and TRAF6 promote MCL-1 K63-linked polyubiquitination on C-terminal lysines. (A) Schematic of MCL-1 lysine mutants. The substituted arginine (R) residues are highlighted in blue. (B) Requirement of the four C-terminal lysine residues in Tax and TRAF6-induced MCL-1 stabilization. Immunoblotting was performed with whole cell lysates of 293 cells transfected with the indicated Flag-MCL-1 plasmids together with Tax or Flag-TRAF6. (C) Identification of C-terminal lysines required for MCL-1 stabilization. Immunoblotting was performed with whole cell lysates of 293 cells either transfected with the indicated Flag-MCL-1 plasmids together with Tax (top two panels) or treated with etoposide for 24 h after transfection (bottom two panels). (D) Ub-independent proteasomal degradation of MCL-1. Immunoblotting was performed with whole cell lysates of 293 cells transfected with Flag-MCL-1 WT or All-KR and then treated with etoposide and/or MG-132 (10 µM) for 24 h. (E) Tax and TRAF6 block the interaction between MCL-1 and the 20S proteasome. Immunoblotting was performed with Flag immunoprecipitates and whole cell lysates derived from 293 cells transfected with Flag-MCL-1 or MCL-1-All-KR together with Tax or His-TRAF6, and treated with the DSP cross-linker (2 µM) for 30 min prior to cell lysis. Asterisk (*) indicates immunoglobulin light chain. (F) Tax promotes the K63-linked polyubiquitination of the four C-terminal MCL-1 lysines. K63-Ub assay was performed with lysates from 293 cells transfected with WT 6×His-MCL-1 and the indicated MCL-1 mutants together with Tax. (G) Proposed molecular mechanism of Tax and TRAF6-induced stabilization of MCL-1.

The E3 ligases MULE, FBW7, and β-TRCP interact with MCL-1 and have been implicated in MCL-1 proteasomal degradation in response to apoptotic stimuli [26], [27], [29]. We next performed co-IP experiments to determine if Tax blocked MCL-1 degradation by inhibiting the interactions between MCL-1 and its degradative E3 ligases. Surprisingly, Tax had no effect on the interaction between MCL-1 and MULE and actually promoted the interactions of MCL-1 with FBW7 and β-TRCP (Figure S15). To further delineate the molecular mechanism underlying MCL-1 stabilization by Tax and TRAF6, we generated a series of compound MCL-1 lysine to arginine mutants (Figures 5A and S16A). A previous study suggested that MCL-1 is ubiquitinated at five N-terminal lysines (amino acids (aa) 5, 40, 136, 194, and 197) to trigger its proteasomal degradation [26]. However, MCL-1 N5-KR, a mutant with the first five lysines substituted with arginine, was still degraded in response to etoposide treatment (Figure S16B), indicating that other lysines may be involved in Ub-dependent proteasomal degradation. Indeed, mutation of the first nine lysines to arginine (N9-KR) abrogated etoposide-induced MCL-1 degradation (Fig. S16B). However, the four C-terminal lysines (aa 276, 279, 302, and 308) were required for Tax and TRAF6-mediated stabilization of MCL-1 and the protection of MCL-1 from etoposide-induced degradation (Figures 5B and C, and S16B-D). These four C-terminal lysines appeared to function in a redundant manner (Figures 5C and S16D). Surprisingly, MCL-1 All-KR (where all lysines were mutated to arginine) was degraded by etoposide in a proteasome-dependent manner since MCL-1 degradation was inhibited by MG-132 treatment (Figure 5D).

Degradation of polyubiquitinated proteins is carried out by the 26S proteasome that includes the core 20S proteasome and a 19S regulatory subunit [39]. Ub-independent degradation of MCL-1 mediated by the 20S proteasome has been previously described [40]. We hypothesized that K63-linked polyubiquitination of MCL-1 impaired its interaction with the proteasome. To test this hypothesis, cells were treated with the DSP (dithiobis[succinimidylpropionate]) cross-linker and lysates were subjected to co-IP experiments to examine the effects of Tax and TRAF6 on MCL-1 interaction with the proteasome. Wild-type MCL-1 interacted with the 20S proteasome as expected, however Tax and TRAF6 effectively blocked this interaction (Figure 5E). Consistently, immunofluorescence experiments showed that MCL-1 colocalized with the 20S proteasome, whereas Tax prevented this colocalization (Figure S17). However, Tax and TRAF6 did not impair interactions between the lysine-less MCL-1 mutant (All-KR) and the proteasome (Figure 5E), indicating a requirement of MCL-1 lysines for Tax/TRAF6 to block MCL-1/20S proteasome binding. Next, ubiquitination assays were performed using wild-type and MCL-1 lysine mutants in order to map the Tax responsive K63-linked polyubiquitination sites in MCL-1. These experiments revealed that the four C-terminal lysines served as the major targets for K63-linked polyubiquitination since MCL-1 C4-KR (and All-KR) was not conjugated with K63-linked polyUb chains in the presence of Tax (Figure 5F). Thus, our collective data indicate that Tax/TRAF6-mediated K63-linked polyubiquitination of the four C-terminal lysines of MCL-1 blocks interactions with the core 20S proteasome (Figure 5G).

### TRAF6 and MCL-1 are essential for the survival of HTLV-1 transformed T cells

**Figure 6:**
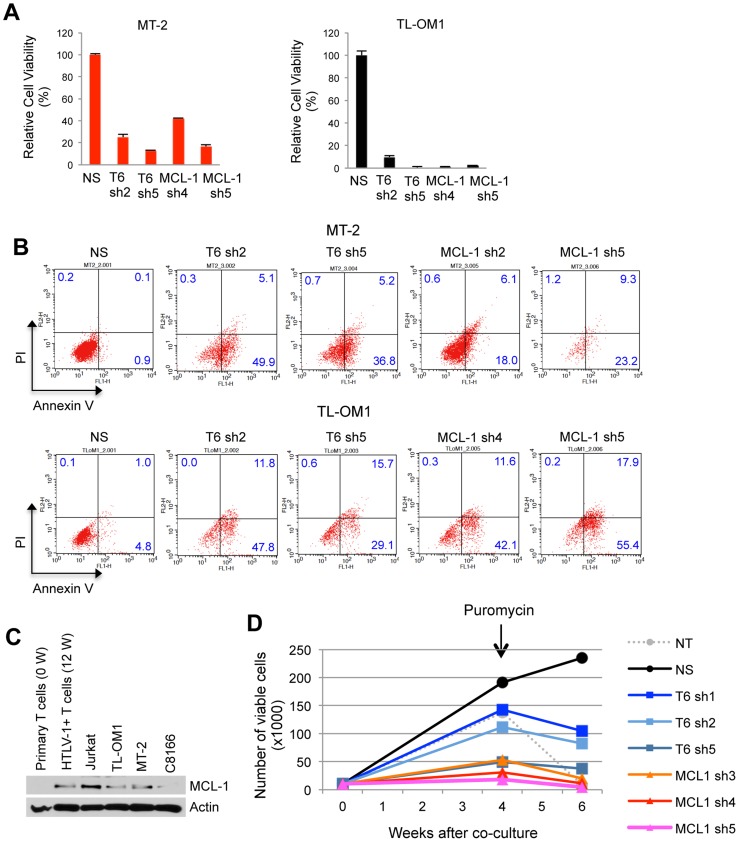
TRAF6 and MCL-1 are required for ATL cell survival and HTLV-1-mediated T-cell immortalization. (A and B) Requirement of TRAF6 and MCL-1 for the viability of HTLV-1 transformed and ATL cell lines. (A) Cell viability assay was performed at 6 days after TL-OM1 and MT-2 cells were transduced with lentiviruses expressing the indicated shRNAs. Relative cell viability (%) was expressed as a percentage relative to the control cells. (B) Flow cytometric analysis of ATL cell lines transduced with shRNAs as described in (A). Cells were stained with both annexin-V-Alexa Fluor 488 and propidium iodide (PI). The distribution of cells is indicated as a percentage in each quadrant. (C) MCL-1 protein is overexpressed in HTLV-1-transformed T cells. Immunoblotting was performed with whole cell lysates of primary human CD4+ T cells (0 W), CD4+ T cells immortalized with HTLV-1 by co-culture with irradiated MT-2 cells for 12 weeks (12 W), Jurkat, ATL (TL-OM1) and HTLV-1 transformed cell lines (MT-2 and C8166). (D) TRAF6 and MCL-1 are essential for the immortalization of T cells by HTLV-1. At four and six weeks after the co-culture of shRNA-transduced PBMCs and irradiated MT-2 cells, viable cells were counted using the trypan blue exclusion method. Puromycin (5 µg/ml) was added at 4 weeks after the co-culture. The data are representative of two independent experiments.

Given the pivotal role of TRAF6 in the control of MCL-1 stability, we hypothesized that TRAF6 plays an essential pro-survival role in ATL cells. Indeed, shRNA-mediated knockdown of TRAF6 resulted in a significant loss of viability of both Tax+ (MT-2) and Tax- (TL-OM1) ATL cells (Figure 6A). Similar results were obtained upon MCL-1 depletion by shRNA (Figure 6A). All shRNAs were validated for specific knockdown of TRAF6 and MCL-1 (Figure S18A and B). Annexin V and propidium iodide (PI) staining confirmed that the decreased viability was due to apoptosis (Figure 6B). Interestingly, MCL-1 depletion in MT-2 cells elicited less cell death compared to TRAF6 depletion (Figure 6B), indicating that the Tax/TRAF6 axis may also activate alternative survival mechanisms, possibly the AKT pathway [35]. Nevertheless, MCL-1 protein expression was significantly elevated in primary human T cells immortalized by HTLV-1 (12 W) using a well-established co-culture assay with irradiated MT-2 cells [41], compared to the parental uninfected primary T cells (0 W) (Figure 6C). These results are congruent with previous studies that demonstrated high levels of MCL-1 protein in HTLV-1 transformed cell lines and immune stimulated Tax expressing cells [42], [43]. To investigate the role of MCL-1 and TRAF6 in HTLV-1-mediated cell transformation, we conducted a co-culture assay using human peripheral blood mononuclear cells (PBMCs) transduced with lentiviruses expressing control scrambled, MCL-1 or TRAF6 shRNAs and lethally irradiated MT-2 cells as a source of infectious HTLV-1 viral particles. Puromycin was added after 4 weeks of co-culture to select for cells containing shRNAs. Cells expressing control shRNAs were immortalized by HTLV-1 and continued to expand indefinitely (Figure 6D). However, cells expressing either TRAF6 or MCL-1 shRNAs ceased to proliferate after 6 weeks in culture and were resistant to immortalization (Figures 6D and S18C), suggesting that TRAF6 and MCL-1 play essential roles in HTLV-1-induced immortalization of primary human T cells.

To determine if TRAF6 played a more ubiquitous role in MCL-1 stabilization and survival of cancer cell lines, TRAF6 was knocked down with shRNA in HeLa (cervical carcinoma), MCF-7 (breast carcinoma), DLD-1 (colorectal adenocarcinoma) and 293 cells. TRAF6 depletion induced the apoptotic death of HeLa, MCF-7 and 293, but not DLD-1 cells, while concomitantly downregulating MCL-1 protein (Figure S19). Thus, TRAF6 regulates the basal stability of MCL-1 in some, but not all cancer cell lines. Knockdown of TRAF6 also sensitized 293 cells to sorafenib-induced cell death (Figure S20). Taken together, TRAF6 regulates MCL-1 stability in diverse cancer cell lines.

## Discussion

Our findings have uncovered a novel mode of regulation of MCL-1 stability that has been hijacked by the HTLV-1 Tax oncoprotein to promote cell transformation. A ubiquitin proteomics screen revealed that Tax modulated the ubiquitination of 136 cellular proteins, of which 22 of these candidates required IKK for Tax-induced ubiquitination. Tax promoted the K63-linked polyubiquitination of MCL-1 in a TRAF6-dependent manner, which imparted enhanced stability to MCL-1 and protection from degradation in response to genotoxic stress stimuli. Our collective results provide strong evidence that Tax has usurped TRAF6 and the host ubiquitin machinery to evade apoptosis and maintain viral persistence.

Although MCL-1 is a highly labile protein, it is commonly overexpressed in cancers and contributes to cell survival and drug resistance although the precise mechanisms have yet to be completely elucidated. Previous studies have shown that the E3 ligases MULE, β-TRCP and FBW7 conjugate MCL-1 with K48-linked polyUb chains to promote its degradation [26]–[29]. The deubiquitinase USP9-X is overexpressed in lymphomas and multiple myeloma and stabilizes MCL-1 by removing K48-linked polyUb chains [44]. Our findings implicate TRAF6 as a key regulator of MCL-1 stability. MCL-1 contains three TRAF6 binding sites in the PEST domain, of which the third one appears to be most critical for binding with TRAF6 and stabilization by Tax. We found that TRAF6 expression was sufficient to protect MCL-1 from etoposide-induced degradation, and TRAF6 required its E3 ligase activity and C-terminal TRAF domain for this function. Intriguingly, treatment of primary mouse B cells with CD40L or LPS, both potent activators of endogenous TRAF6, protected MCL-1 from etoposide-induced degradation. We also found that TRAF6 stabilized MCL-1 in mouse fibroblasts and in several cancer cell lines including HeLa and MCF-7. Therefore, TRAF6-mediated MCL-1 stabilization appears to be a common mechanism of cell survival usurped by both viral and non-viral cancers.

Tax contains a TRAF6 binding motif between amino acids 343–348, just upstream of the PBM known to be critical for transformation by Tax [32]. Mutation of this TRAF6 binding site greatly diminished Tax interaction with TRAF6, Tax-induced autoubiquitination of TRAF6 and MCL-1 K63-linked polyubiquitination and stabilization. Tax interaction with TRAF6 was also critical for the redistribution of TRAF6 to the mitochondria since Tax^E345A^ was defective in promoting TRAF6 mitochondrial localization. Thus, Tax directly engages TRAF6 to trigger its activation, and a subset of Tax translocates to the mitochondria together with TRAF6 which directly ubiquitinates MCL-1 and possibly other substrates. It is intriguing that Tax can localize to the mitochondria, however future studies will be necessary to determine the precise mechanisms by which Tax traffics to the mitochondria. Notably, the Tax2 protein encoded by HTLV-2 has divergent C-terminal sequences from Tax1, lacks the TRAF6 binding site and PBM and is unable to interact with TRAF6 [45]. These differences in Tax2 may potentially account for the reduced pathogenicity of HTLV-2, which has not been definitively linked to any lymphoproliferative disorders [46].

Previous studies have shown that Tax enhances the autoubiquitination of TRAF6 [30], consistent with the findings in this report. Given that TRAF6 is a potent activator of NF-κB, does TRAF6 play a role in Tax-induced NF-κB activation? A previous study demonstrated that Tax activation of IKK proceeds normally in the absence of TRAF6 *in vitro* using a cell free assay system [47]. However, our findings in this study raise new questions regarding TRAF6 and Tax-induced NF-κB activation that clearly warrant additional studies performed in T cells, the natural cell host of HTLV-1. Indeed, we have recently found that IL-17RB and TRAF6 both play essential roles in NF-κB signaling in HTLV-1 transformed cell lines [48]. However, it is unclear if Tax requires TRAF6 and the TRAF6 binding site in Tax for NF-κB activation. It also remains unclear precisely how IKK participates in Tax-induced TRAF6 activation and MCL-1 stabilization. Since Tax forms protein complexes with IKK/NEMO and TRAF6, Tax may utilize NEMO as part of its mechanism to trigger TRAF6 autoubiquitination. Given that Tax also induces the mitochondrial localization of IKK, another possibility is that IKK may directly phosphorylate and regulate either TRAF6 and/or MCL-1 in the mitochondria.

Our results indicated that Tax did not impede the interactions of MCL-1 with its degradative E3 ligases, but rather Tax enhanced binding of MCL-1 with FBW7 and β-TRCP. Consistently, Tax also modestly increased the K48-linked polyubiquitination of MCL-1. These findings can potentially be explained by a recent study that demonstrated that Tax induces reactive oxygen species, which in turn stimulate DNA damage and genotoxic stress [49]. Tax may counteract the destabilizing effects of genotoxic stress on MCL-1 by triggering the activation and mitochondrial localization of TRAF6, which interacts with MCL-1 and conjugates the four C-terminal lysine residues with K63-linked polyUb chains. MCL-1 K63-linked polyubiquitination stabilizes MCL-1 by blocking its interactions with the core 20S proteasome, thereby preventing both Ub-dependent and independent degradation of MCL-1. K63-linked polyUb chains conjugated to the C-terminus of MCL-1 may prevent 20S proteasome binding via a conformational change in MCL-1 or through steric hindrance.

Previously it was reported that pro-apoptotic BH3-only proteins (BOPs), PUMA and BIM, stabilize yet inactivate MCL-1 [50]–[52]. This paradoxical finding indicates that functional inactivation of MCL-1 does not always require its degradation. Although the molecular mechanism remains to be elucidated, PUMA and BIM may stabilize MCL-1 by binding to the hydrophobic groove of MCL-1 to inhibit MULE interactions [51]. However, our results indicate that Tax did not prevent MCL-1 and MULE interactions, and also TRAF6 did not bind to the hydrophobic groove of MCL-1. Therefore, it is likely that Tax and TRAF6 stabilize and enhance the anti-apoptotic activity of MCL-1, in contrast to PUMA and BIM. This notion is further supported by our results that demonstrate that shRNA-mediated knockdown of MCL-1 in HTLV-1-transformed and ATL cells induced apoptotic cell death and also blocked HTLV-1-induced immortalization of primary T cells.

TRAF6 has recently emerged as an oncogene and is overexpressed in diverse human cancers [53], [54]. TRAF6 conjugates AKT with K63-linked polyUb chains that regulate its membrane localization and phosphorylation [35]. TRAF6 also upregulates the expression of hypoxia-inducible factor 1α (HIF-1α) to promote tumor angiogenesis [55]. Together with our findings that TRAF6 governs MCL-1 stability and cell survival, accumulating evidence strongly support the notion that TRAF6 is a bona fide oncogene and when overexpressed can endow cells with at least three of the known hallmarks of cancer (sustaining proliferative signaling, resisting cell death and inducing angiogenesis) [56]. In conclusion, our studies have identified a novel TRAF6/MCL-1 anti-apoptotic axis that has been subverted by the HTLV-1 Tax oncoprotein to evade apoptosis. TRAF6 and MCL-1 may therefore represent viable therapeutic targets for ATL and other cancers.

## Materials and Methods

### Ethics statement

Blood from healthy donors was purchased from Biological Specialty Corporation (Colmar, PA).

Animal work was carried out in strict accordance with the recommendations in the Guide for the Care and Use of Laboratory Animals of the National Institutes of Health. The animal protocol (protocol number MO12M112) was approved by the Institutional Animal Care and Use Committee (IACUC) of Johns Hopkins University.

### Plasmids

pCMV-Flag-MCL-1 (plasmid 25392), pRK5-HA-Ub (plasmid 17608), pRK5-HA-Ub-K63 (plasmid 17606) and pRK5-HA-Ub-K48 (plasmid 17605) were purchased from Addgene. His-MCL-1 was generated by cloning human MCL-1 cDNA into pcDNA3.1/His using BamHI and EcoRI enzyme sites. pCMV4-Tax and M22 were previously described [20]. Flag-Tax (wild-type and mutants) was generated by cloning Tax cDNAs into pcDNA3.1/Flag vector using BamHI and EcoRI enzyme sites. The plasmids expressing HA-TRAF3, Flag-TRAF2 and Flag-TRAF6 were previously described [57]–[59]. Flag-TRAF5 [60] was a gift from Drs. Soo Young Lee and Yongwon Choi (University of Pennsylvania). Flag-TRAF3 was generated by cloning TRAF3 cDNA into pDUET.Hyg vector using BamHI and XhoI enzyme sites. Myc/His-TRAF6 was generated by cloning TRAF6 cDNA into pcDNA3.1-Myc/His vector using EcoRI and XhoI enzyme sites. The GFP reporter vector pEGFP N1 was purchased from Clontech. GFP-Tax [61] was a gift from Dr. Brian Wigdahl (Drexel University). Flag-EBV LMP1 plasmid [62] was a gift from Dr. Shunbin Ning (University of Miami). Flag-MULE [26] was a gift from Dr. Qing Zhong (University of California, Berkeley). HA-FBW7 plasmid [28] was a gift from Dr. Wenyi Wei (Harvard University). HA-βTRCP [63] was a gift from Dr. Shao-Cong Sun (M.D. Anderson Cancer Center). Tax, IKKα, and IKKβ shRNAs were cloned into lentiviral pYNC352/puro or GFP-puro vector using BamHI and MluI enzyme sites as described previously [64]. The oligonucleotide sequences for shRNAs are listed in Table S2. The lentiviral pLKO.puro vector expressing control, TRAF6 and MCL-1 shRNAs were purchased from Sigma-Aldrich. For lentiviral transduction of Tax, Tax cDNA was cloned into pDUET.hyg using BamHI and XhoI enzyme sites. GST-MCL-1 was generated by cloning MCL-1 cDNA lacking the C-terminal transmembrane domain (amino acids 328 to 350) in frame with GST into pGEX4T-1 using BamHI and XhoI enzyme sites. Site-directed mutagenesis was performed using Platinum Pfx DNA polymerase (Invitrogen).

### Antibodies

Anti-MCL-1 (sc-819), TRAF6 (sc-8409), TOM20 (sc-17764), GFP (sc-8334), GST (sc-138), His (sc-804), VDAC1 (sc-390996), IKKα (sc-7182), IKKβ (sc-8014), NEMO (sc-8330) and LDH (sc-33781) antibodies were purchased from Santa Cruz Biotechnology (Santa Cruz, CA). Anti-mouse MCL-1 antibody (600-401-394) was purchased from Rockland Immunochemicals (Gilbertsville, PA). Anti-TRAF6 (8028), Flag (2368), PARP (9532), and Lys63 specific ubiquitin (5621) antibodies were purchased from Cell Signaling Technology (Danvers, MA). Anti-ubiquitin (linkage-specific K63) antibody (ab179434) was from Abcam (Cambridge, MA). Anti-Flag (F1804) and β-actin (A1978) antibodies were purchased from Sigma-Aldrich (St. Louis, MO). Anti-Myc (OP-10) and Lys63 specific ubiquitin (05-1313) monoclonal antibodies were purchased from EMD Millipore (Billerica, MA). Anti-ubiquitin (ADI-SPA-200) and 20S proteasome α4 antibodies (MCP34) were purchased from ENZO Life Sciences (Farmingdale, NY). Anti-HA (clone 12CA5) antibody was purchased from Roche Applied Science (Indianapolis, IN). Monoclonal anti-Tax antibody was prepared from a hybridoma (168B17-46-34) received from the AIDS Research and Reference Program, NIAID, National Institutes of Health.

### Cell culture, transfections, and lentiviral transductions

Jurkat Tet/On-Tax WT and M22 cells [65] were maintained in RPMI 1640 supplemented with 10% tetracycline-free FBS and treated with 1 µg/ml doxycycline for 2 days for inducible Tax expression. Jurkat, Jurkat (NEMO-deficient) [66], DLD-1, and HTLV-1 transformed and ATL cell lines including MT-2, MT-4, SLB-1, ATL-2(S), C8166, TL-OM1, ED40515(-) and ATL-43T were cultured in RPMI-1640 supplemented with 10% heat-inactivated FBS and antibiotics. HeLa, 293T, 293, MCF-7 and Raw 264.7 cell lines were cultured in DMEM supplemented with 10% FBS and antibiotics. *Traf6^+/+^* and *Traf6^−/−^* MEFs, a gift from Dr. Hui-Kuan Lin (M.D. Anderson Cancer Center), were cultured in DMEM supplemented with 10% FBS and antibiotics and transfected with GenJet II (SignaGen Laboratories, Rockville, MD). Primary splenic B cells were isolated using the EasySep mouse B cell enrichment kit (Stemcell Technologies, Vancouver, Canada) from mice on a mixed 129×B6 genetic background and cultured in RPMI-1640 supplemented with 10% heat-inactivated FBS. Plasmid DNA transfection of HeLa, 293 and 293T cells was performed using JetPrime (Polyplus-Transfection, New York, NY). Lentiviral transduction of DNA or short hairpin RNAs (shRNAs) into suspension cells was performed by spinoculation at 800×g for 30 min with MOIs of 5 to 10. The lentiviral transduction of adherent cells was conducted in the presence of 5 µg/ml polybrene.

### Immunoblotting, immunoprecipitation, and immunostaining

For immunoblotting, whole cell lysates were prepared by lysing cells in cell extraction buffer (100 mM Tris [pH 7.4], 100 mM NaCl, 1% Triton X-100, 1 mM EDTA, 1 mM EGTA, 10% glycerol, 0.1% SDS, 2 mM Na_2_VO_4_, 1 mM NaF, 0.5% deoxycholate, and 20 mM Na_4_P_2_O_7_) supplemented with protease inhibitor cocktail (Roche Applied Bioscience, Indianapolis, IN) on ice, followed by centrifugation at 15,000×g for 10 min. Cell lysates were separated on SDS-PAGE, transferred to nitrocellulose membranes and immunoblotted with appropriate antibodies diluted in SuperBlock blocking PBS buffer (Thermo Scientific, Rockford, IL). For co-IP, cells were lysed in RIPA buffer (50 mM Tris [pH 7.4], 150 mM NaCl, 1% Igepal CA-630, and 0.25% deoxycholate) freshly supplemented with protease inhibitor cocktail on ice. Lysates (500 µg protein) cleared by centrifugation were incubated at 4°C overnight with the indicated antibodies (2 µg) and then incubated with protein A-agarose beads for an additional 3 h. Immunoprecipitates were washed three times with RIPA buffer followed by elution of bound proteins with 1.5× SDS sample buffer or 3× Flag peptide (Sigma, St. Louis, MO). For immunostaining, HeLa cells grown on glass coverslips for 24 h were transfected, and fixed and permeabilized in chilled methanol for 5 min. For staining of mitochondria, cells were incubated with 100 nM Red MitoTracker (Invitrogen, Grand Island, NY) for 30 min before fixation. Following incubation with SuperBlock blocking PBS buffer overnight at 4°C, coverslips were incubated with primary antibodies, washed with PBS, and then incubated with appropriate fluorescence dye-conjugated secondary antibodies. The coverslips were incubated with DAPI for 3 min and mounted in mounting medium (Richard-Allan Scientific, Campus Drive Kalamazoo, MI), and cells were imaged on Nikon E-800 with a 60× oil-corrected objective.

### UbiScan proteomics screen

The UbiScan proteomics platform (performed by Cell Signaling Technology) was used to identify and quantify differences in ubiquitination between untreated Jurkat Tet/On-Tax WT and M22 cells and treated with Dox. Briefly, cell lysates were digested with trypsin and peptides were separated from non-peptide material by solid phase extraction with Sep-Pak C18 cartridges. Lyophilized peptides were re-dissolved, and ubiquitinated peptides enriched with the ubiquitin branch (“K-ε-GG”) antibody (Cell Signaling Technology). Peptides were eluted from antibody resin into a total volume of 100 µl in 0.15% TFA. Eluted peptides were concentrated with Eppendorf PerfectPure C18 tips prior to LC-MS/MS analysis with an LTQ-Orbitrap hybrid mass spectrometer. MS/MS spectra were evaluated using SEQUEST and SORCERER 2. Peptide assignments were obtained using a 5% false positive discovery rate. Searches were performed against the NCBI human database.

### Protein ubiquitination assays

For ubiquitination assays, an extra wash was performed using RIPA buffer supplemented with 1 M urea after immunoprecipitation. For MCL-1 ubiquitination assays performed with His-MCL-1, cells were lysed in buffer B (100 mM NaH_2_PO_4_, 10 mM Tris, and 8 M urea [pH 8.0]) and His-tagged MCL-1 proteins were precipitated with Ni-nitrilotriacetic acid (NTA) agarose (Qiagen, Valencia, CA). After washing in buffer C (100 mM NaH_2_PO_4_, 10 mM Tris, and 8 M urea [pH 6.3]), His-tagged proteins were eluted in buffer E (100 mM NaH_2_PO_4_, 10 mM Tris, and 8 M urea [pH 4.5]) and subjected to SDS-PAGE and immunoblotting. For TRAF6 *in vitro* ubiquitination assays, Flag-tagged TRAF6 or Tax proteins expressed in 293T cells were purified using EZview Red anti-Flag affinity gel (Sigma-Aldrich, St. Louis, MO) and incubated in ubiquitin conjugation reaction buffer supplemented with UBE1 (E-305), UbcH13 (E2-664) and ubiquitin (U-100H) purchased from Boston Biochem (Cambridge, MA) for 2 h at 30°C. For MCL-1 *in vitro* ubiquitination assays, Flag-TRAF6 (alone or with Tax) was purified and incubated in ubiquitin conjugation reaction buffer supplemented with UBE1, UbcH5c (E2-627), GST or GST-MCL-1, ubiquitin and energy regeneration buffer (Boston Biochem) for 2 h at 30°C. The reaction mixtures were boiled in 1× SDS sample buffer and subjected to SDS-PAGE and immunoblotting.

### *In vitro* protein binding assay

Recombinant GST-fusion proteins were purified using standard methods. To enhance the solubility of GST-MCL-1 proteins from bacteria, the C-terminal transmembrane domain of MCL-1 was deleted. GST-MCL-1 protein (2 µg) immobilized on glutathione beads was incubated with Flag-TRAF6 protein (500 ng) immunoprecipitated from 293T cells in binding buffer (50 mM Tris [pH 7.4], 150 mM NaCl, 1 mM EDTA, and 1% Triton X-100) for 3 h at 4°C and washed with binding buffer four times, and precipitated protein complexes were separated on SDS-PAGE and subjected to immunoblotting.

### Cycloheximide chase assay

Cells were treated with CHX (10 µg/ml) for the indicated times. Whole cell lysates were prepared in cell extraction buffer, separated on SDS-PAGE, and immunoblotted with anti-MCL-1 and actin antibodies. The intensity of MCL-1 bands was measured using Alphaview software (Alpha Innotech, San Leandro, CA) and normalized to actin. The half-life of MCL-1 protein was calculated under the assumption of first-order decay kinetics as described previously [67].

### Subcellular fractionation

Mitochondria were isolated using Axis-Shield OptiPrep iodixanol (Sigma-Aldrich, St. Louis, MO) according to the manufacturer's protocol. Briefly, cells transfected with the indicated plasmid DNAs were homogenized in buffer B (0.25 M sucrose, 1 mM EDTA, 20 mM HEPES-NaOH [pH 7.4]) with 40 strokes of a Dounce homogenizer and centrifuged at 1,000 g for 10 min. An aliquot of homogenate was used as total cell extract. The supernatant was further centrifuged at 13,000 g at 4°C for 10 min. The supernatant was used as cytosol fraction. The pellet resuspended in 36% iodixanol was bottom-loaded under 10% and 30% gradients and centrifuged at 50,000 g for 4 h. The mitochondrial fraction was collected at the 10%/30% iodixanol interface.

### Quantitative real time-PCR (qRT-PCR)

Total RNA was isolated using the RNeasy mini kit (Qiagen, Valencia, CA). First-strand cDNA was synthesized from 1 µg of total RNA using SuperScript II RT (Invitrogen) with random hexamers. qRT-PCR was performed in a 96-well microplate using an ABI Prism 7500 detection system (Applied Biosystems, Foster City, CA) with the RT^2^Real-Time SYBR green/ROX master mix (Qiagen, Valencia, CA). Reactions were performed in a total volume of 25 µl and contained 50 ng of reverse-transcribed RNA (based on the initial RNA concentration) and gene-specific primers. PCR conditions included an initial incubation step of 2 min at 50°C and an enzyme heat activation step of 10 min at 95°C, followed by 40 cycles of 15 seconds at 95°C for denaturing and 1 min at 60°C for annealing and extension. Primer sequences are listed in Table S3.

### HTLV-1 immortalization co-culture assay

Peripheral blood mononuclear cells (PBMCs) were freshly isolated from blood using Ficoll-Paque Plus (GE healthcare, Piscataway, NJ) and stimulated with 5 µg/ml of phytohemagglutinin (PHA) in R10 media (RPMI-1640 supplemented with 25 mM HEPES, 2 mM L-glutamine, and antibiotics) for 3 days. Following transduction (MOI of 10) with lentiviruses expressing shRNAs by spinoculation at 800×g for 3.5 h, the PBMCs (1×10^4^ cells) were co-cultured with MT-2 cells (5×10^4^ cells) that were γ-irradiated at 60 Gy. Puromycin (5 µg/ml) was added to the culture at 4 week after co-culture to select for shRNA-transduced PBMCs. Viable cells were counted using the trypan blue exclusion assay at 4 and 6 weeks.

### Cell apoptosis and viability assays

To measure apoptosis, a modified Annexin V/PI staining procedure was performed as described previously [68]. Briefly, cells were washed in phosphate buffered saline (PBS) and 1× Annexin V binding (AV) buffer (10 mM HEPES [pH 7.4], 140 mM NaCl, and 2.5 mM CaCl_2_) twice, and resuspended in 100 µl of AV buffer. The cells were incubated with Annexin V Alexa Fluor 488 (Invitrogen) in the dark for 15 min. at room temperature and then PI (Sigma-Aldrich) was added for an additional 15 min, fixed with 1% formaldehyde and treated with RNase A (50 µg/ml) for 15 min at 37°C. Samples were analyzed using a FACSCalibur flow cytometer (BD Biosciences). CellTiter-Glo Luminescent Cell Viability Assay (Promega, Madison, WI) which quantitates ATP as a measure of metabolically active cells was used to measure cell viability.

## Supporting Information

Figure S1 **Tax induces the K63-linked polyubiquitination of MCL-1.** Ubiquitination assay was performed by MCL-1 immunoblotting of HA-immunoprecipitates (IPs) derived from 293T cells transfected with MCL-1 together with HA-Ub (WT, K48-only, and K63-only) in the presence or absence of Tax (top panel). “Input” indicates HA-immunoblotting of 5% of the 293T whole cell lysates used in the immunoprecipitation (bottom panel).
(PDF)

Figure S2 **Tax specifically induces the K63-linked polyubiquitination of MCL-1.** Ubiquitination assay was performed with immunoprecipitates (IPs) derived from 293T cells transfected with His-MCL-1, with or without Tax. Due to enhanced stabilization of MCL-1 by Tax in lysates, the lysate volume was adjusted to ensure equal amounts of MCL-1 for the IP.
(PDF)

Figure S3 **Tax promotes TRAF6 autoubiquitination.** *In vitro* ubiquitination assay was performed in the presence or absence of ATP with Flag-immunoprecipitates derived from 293T cells transfected with Flag-TRAF6 WT or C70A together with empty vector (EV) or Tax. The reaction mixtures were separated on SDS-PAGE and immunoblotted with anti-Ub (top panel) or Flag antibodies (middle panel). “Input” indicates Tax-immunoblotting of 5% of the 293T whole cell lysates used in the IP (bottom panel).
(PDF)

Figure S4 **TRAF6 conjugates MCL-1 with polyubiquitin chains.** *In vitro* ubiquitination assay was performed with Flag TRAF6-immunoprecipitates derived from 293T cells transfected with or without Tax, washed with 1× ubiquitin reaction buffer and incubated with 50 nM E1 enzyme (UBE1), 80 nM E2 enzyme (UbcH5c), 500 µM ubiquitin, energy regeneration solution and 2 µg of recombinant GST or GST-MCL-1 for 2 h at 30°C. The reaction was terminated upon boiling in sample buffer and the reaction mixtures were separated by SDS-PAGE and immunoblotted with anti-GST.
(PDF)

Figure S5 **Tax induces the mitochondrial localization of TRAF6.** Immunoblotting was performed with whole cell homogenates (Total) and mitochondrial fractions (Mito) derived from 293T cells transfected with Flag-TRAF2 (**A**), HA-TRAF3 (**B**), Flag-TRAF5 (**C**), and Flag-TRAF6 (**D**), in the presence or absence of Tax. Fifty fold excess of mitochondrial extracts over total cell homogenates was loaded onto the gel to achieve near normalization. TOM20 was used as a marker for mitochondria.
(PDF)

Figure S6 **Tax requires the C-terminal TRAF domain of TRAF6 for its mitochondrial localization.** Immunofluorescence assay was performed with HeLa cells transfected with Flag-TRAF6^ΔTRAF-C^ together with Tax, Tax^M22^ or Tax^E345A^ and incubated with MitoTracker Red for 30 min before fixation. Tax and TRAF6 were stained with Alexa Fluor 647 (artificially colored purple) and Alexa Flour 488 (green), respectively. Nuclei were counterstained with DAPI (blue) before mounting coverslips.
(PDF)

Figure S7 **Tax requires NEMO for MCL-1 stabilization.** Cycloheximide chase assays were performed by immunoblotting with whole cell lysates derived from wild-type and NEMO-deficient Jurkat cells lentivirally transduced with GFP or Tax at the indicated times after cycloheximide treatment (10 µg/ml).
(PDF)

Figure S8 **Tax protects MCL-1 from genotoxic stress-induced degradation.** **(A)** Immunoblotting was performed with whole cell lysates derived from Jurkat Tet/On-Tax cells cultured in the presence or absence of doxycycline (Dox, 1 µg/ml) for 48 h followed by UV-irradiation (200 J/m^2^). **(B)** Immunoblotting was performed with whole cell lysates derived from TL-OM1 and MT-2 cells treated with cisplatin (25 µM), daunorubicin (5 µM), etoposide (10 µg/ml) and sorafenib (10 µM) for 24 h.
(PDF)

Figure S9 **IKK protects MCL-1 from etoposide-induced degradation in HTLV-1 transformed cells.** (**A** and **B**) Immunoblotting was performed with whole cell lysates derived from MT-2 cells lentivirally transduced with shRNAs specific for IKKα and IKKβ for 3 days and treated with etoposide (10 µg/ml) for 24 h. **(C)** Immunoblotting was performed with whole cell lysates derived from MT-2 cells pretreated with IKKβ inhibitor SC-514 (20 µM) for 1 h and treated with etoposide for 24 h. **(D)** Immunoblotting was performed with the indicated fractions derived from Jurkat Tet/On-Tax cells either uninduced or induced with Dox for 48 h. The cells were treated with etoposide (10 µM) as indicated for 6 h before harvesting. A fifty-fold excess of mitochondrial extracts (M) compared to total cell homogenates (T) were loaded for normalization.
(PDF)

Figure S10 **Tax does not transcriptionally regulate MCL-1.** qRT-PCR analysis was performed using gene-specific primers with total RNAs isolated from Jurkat Tet/On-Tax cultured with Dox for 0, 1 and 2 days. Graphs depict fold change of mRNA expression relative to cells at 0 days.
(PDF)

Figure S11 **Tax does not regulate MCL-1 mRNA expression in HTLV-1 transformed cell lines.** qRT-PCR analysis was performed for the indicated genes with total RNAs isolated from Jurkat, HTLV-1 transformed and ATL cell lines including ED40515(-), TL-OM1, MT-2 and C8166. Graphs depict fold change of mRNA expression relative to Jurkat cells.
(PDF)

Figure S12 **CD40L and LPS do not transcriptionally activate MCL-1 in primary murine B cells.** qRT-PCR analysis was performed using gene-specific primers for MCL-1 (A), ICAM-1 (B) and A20 (C) with total RNAs isolated from primary splenic B cells treated with CD40L (100 ng/ml) and LPS (100 ng/ml) for 6 h. Graphs depict fold change of mRNA expression relative to untreated cells.
(PDF)

Figure S13 **EBV LMP1 protects MCL-1 from etoposide-induced degradation.** Immunoblotting was performed with whole cell lysates derived from 293T cells transfected with Flag-MCL-1 together with empty vector or Flag-LMP1 for 24 h and treated with etoposide as indicated for an additional 24 h.
(PDF)

Figure S14 **TRAF6 is required for LPS-induced MCL-1 ubiquitination.** **(A)** Ubiquitination assay was performed by IP with α-MCL1 from lysates of RAW 264.7 cells left untreated or treated with LPS (100 ng/ml) for 24 h. Immunoblotting was performed using anti-Ub and K63 Ub antibodies. **(B)** Ubiquitination assay was performed by IP with α-MCL1 from lysates of RAW 264.7 cells lentivirally transduced with an shRNA targeting mouse TRAF6 for 3 days and treated with LPS (100 ng/ml) for an additional 24 h, and immunoblotting with anti-K63 Ub antibody.
(PDF)

Figure S15 **Tax does not impair the interactions between MCL-1 and its degradative E3 Ub ligases.** Co-IP was performed with whole cell lysates derived from 293T cells transfected with Flag-MCL-1 together with Flag-MULE (**A**), HA-FBW7 (**B**) or HA-βTRCP (**C**), in the presence or absence of Tax. The cells were left untreated or treated with etoposide (10 µM) for 6 h in the presence of MG-132 (10 µM) before harvesting.
(PDF)

Figure S16 **Mapping of MCL-1 lysine residues required for its etoposide-induced degradation.** **(A)** Illustration of MCL-1 lysine to arginine mutants. The substituted arginine (R) residues are highlighted in blue. Immunoblotting was performed with whole cell lysates derived from 293 cells transfected with Flag-MCL-1 mutants in which lysine residues were sequentially replaced with arginine mutated from the N-terminus (**B**) or C-terminus (**C**), and left untreated or treated with etoposide (10 µM) for 24 h. **(D)** Immunoblotting was performed with whole cell lysates of 293 cells transfected with the indicated Flag-MCL-1 plasmids and treated with etoposide for 24 h after transfection.
(PDF)

Figure S17 **Tax inhibits the co-localization of MCL-1 and the core 20S proteasome (α4 subunit).** Immunofluorescence assay was performed with HeLa cells transfected with Flag-MCL-1 together with GFP or GFP-Tax. MCL-1 and 20S were stained with Alexa Fluor 647 (red) and Alexa Fluor 594 (artificially colored green), respectively. The GFP fluorescence signal was changed to gray scale. Nuclei were counterstained with DAPI (blue) before mounting coverslips.
(PDF)

Figure S18 **Depletion of TRAF6 and MCL-1 prevent HTLV-1-mediated T-cell immortalization. (A and B)** Immunoblotting was performed with whole cell lysates derived from 293T cells transfected for 3 days with TRAF6 (**A**) or MCL-1 shRNAs (**B**). **(C)** Co-culture assay with PBMCs transduced with the indicated shRNAs and irradiated MT-2 cells. Bright field images of the central area of each well were taken 6 weeks after co-culture at 10× magnification.
(PDF)

Figure S19 **Depletion of TRAF6 attenuates cell growth and promotes apoptotic cell death of cancer cell lines. (A–D)** Cell lines including HeLa (**A**), MCF-7 (**B**), DLD-1 (**C**) and 293 (**D**) were lentivirally transduced with TRAF6 shRNAs, and cell growth was measured by counting the number of viable cells using trypan blue exclusion at the indicated days. Immunoblotting was performed with whole cell lysates of the cells at 3 days after lentiviral transduction using the indicated antibodies.
(PDF)

Figure S20 **Knockdown of TRAF6 sensitizes cells to sorafenib-induced death.** 293 cells were lentivirally transduced with control NS shRNA(A) and TRAF6 shRNAs 1 (B), 2 (C) and 5 (D) and 2 days later treated with sorafenib (1 µM) for 24 h, and bright field microscopic images were taken at 10× magnification.
(PDF)

Table S1 **Ubiquitin proteomics results from Jurkat cells inducibly expressing Tax or Tax M22.** Summary table of cellular proteins with ubiquitination sites modulated by Tax and/or Tax M22. Green highlight indicates a 2.5-fold or more induction of the indicated ubiquitination site by Tax or Tax M22. Red highlight indicates at least a 2.5-fold decrease of the indicated ubiquitination site by Tax or Tax M22. The protein name is in bold if ubiquitinated in an IKK-dependent manner (by Tax WT but not Tax M22).
(PDF)

Table S2 **Oligonucleotide sequences for shRNAs.**
(PDF)

Table S3 **Primer sequences for qRT-PCR.**
(PDF)
