# Supplementary figures and images for: HTLV-1 Tax Stabilizes MCL-1 via TRAF6-Dependent K63-Linked Polyubiquitination to Promote Cell Survival and Transformation
Source: PLoS Pathog. 2014 Oct 23;10(10):e1004458. doi: 10.1371/journal.ppat.1004458 (PMC4207805; doi:10.1371/journal.ppat.1004458)

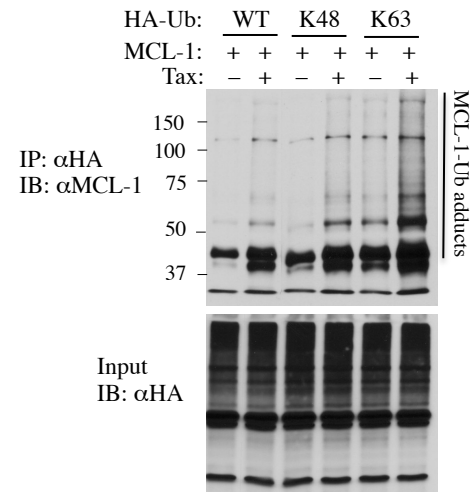

Supplement: Figure S1 — Tax induces the K63-linked polyubiquitination of MCL-1. Ubiquitination assay was performed by MCL-1 immunoblotting of HA-immunoprecipitates (IPs) derived from 293T cells transfected with MCL-1 together with HA-Ub (WT, K48-only, and K63-only) in the presence or absence of Tax (top panel). “Input” indicates HA-immunoblotting of 5% of the 293T whole cell lysates used in the immunoprecipitation (bottom panel). (PDF) [file ppat.1004458.s001.pdf]

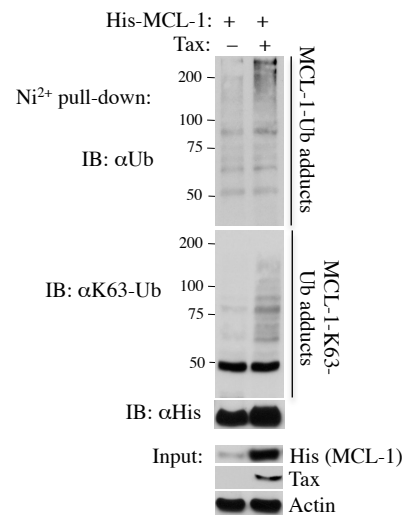

Supplement: Figure S2 — Tax specifically induces the K63-linked polyubiquitination of MCL-1. Ubiquitination assay was performed with immunoprecipitates (IPs) derived from 293T cells transfected with His-MCL-1, with or without Tax. Due to enhanced stabilization of MCL-1 by Tax in lysates, the lysate volume was adjusted to ensure equal amounts of MCL-1 for the IP. (PDF) [file ppat.1004458.s002.pdf]

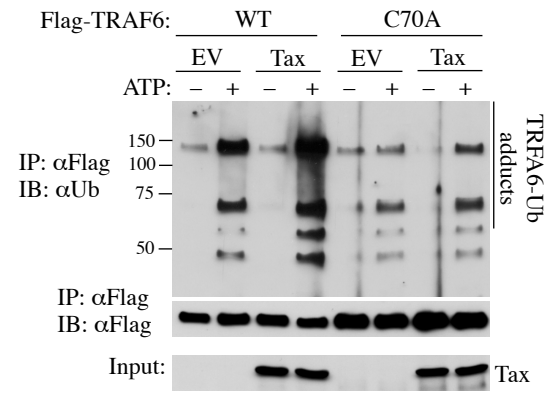

Supplement: Figure S3 — Tax promotes TRAF6 autoubiquitination. In vitro ubiquitination assay was performed in the presence or absence of ATP with Flag-immunoprecipitates derived from 293T cells transfected with Flag-TRAF6 WT or C70A together with empty vector (EV) or Tax. The reaction mixtures were separated on SDS-PAGE and immunoblotted with anti-Ub (top panel) or Flag antibodies (middle panel). “Input” indicates Tax-immunoblotting of 5% of the 293T whole cell lysates used in the IP (bottom panel). (PDF) [file ppat.1004458.s003.pdf]

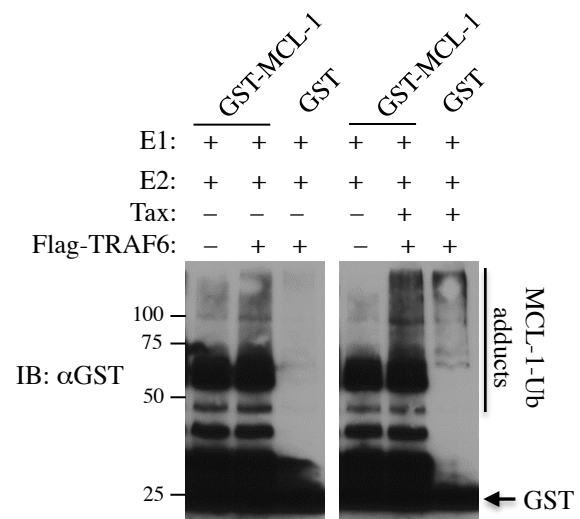

Supplement: Figure S4 — TRAF6 conjugates MCL-1 with polyubiquitin chains. In vitro ubiquitination assay was performed with Flag TRAF6-immunoprecipitates derived from 293T cells transfected with or without Tax, washed with 1× ubiquitin reaction buffer and incubated with 50 nM E1 enzyme (UBE1), 80 nM E2 enzyme (UbcH5c), 500 µM ubiquitin, energy regeneration solution and 2 µg of recombinant GST or GST-MCL-1 for 2 h at 30°C. The reaction was terminated upon boiling in sample buffer and the reaction mixtures were separated by SDS-PAGE and immunoblotted with anti-GST. (PDF) [file ppat.1004458.s004.pdf]

**A**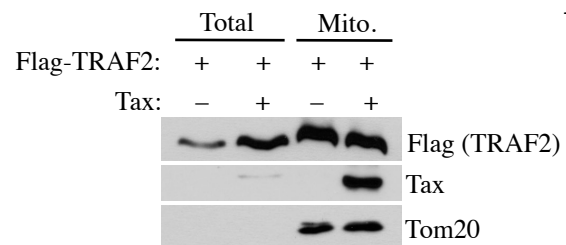**B**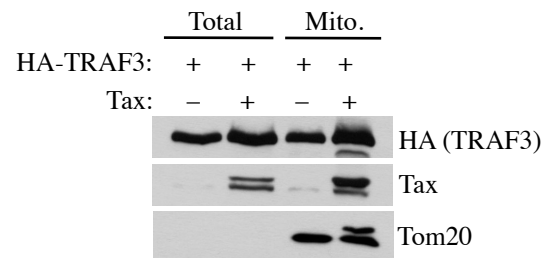**C**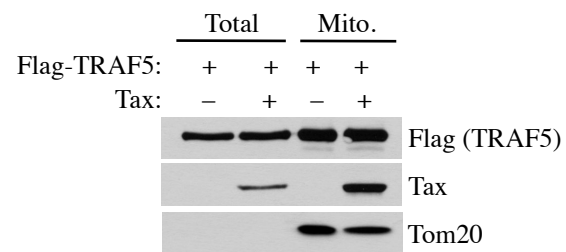**D**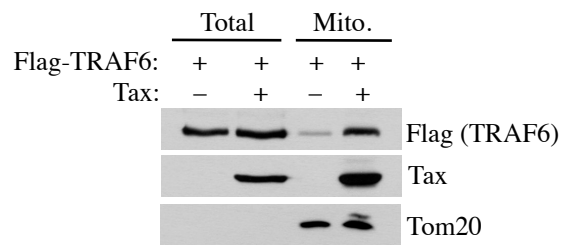

Supplement: Figure S5 — Tax induces the mitochondrial localization of TRAF6. Immunoblotting was performed with whole cell homogenates (Total) and mitochondrial fractions (Mito) derived from 293T cells transfected with Flag-TRAF2 (A), HA-TRAF3 (B), Flag-TRAF5 (C), and Flag-TRAF6 (D), in the presence or absence of Tax. Fifty fold excess of mitochondrial extracts over total cell homogenates was loaded onto the gel to achieve near normalization. TOM20 was used as a marker for mitochondria. (PDF) [file ppat.1004458.s005.pdf]

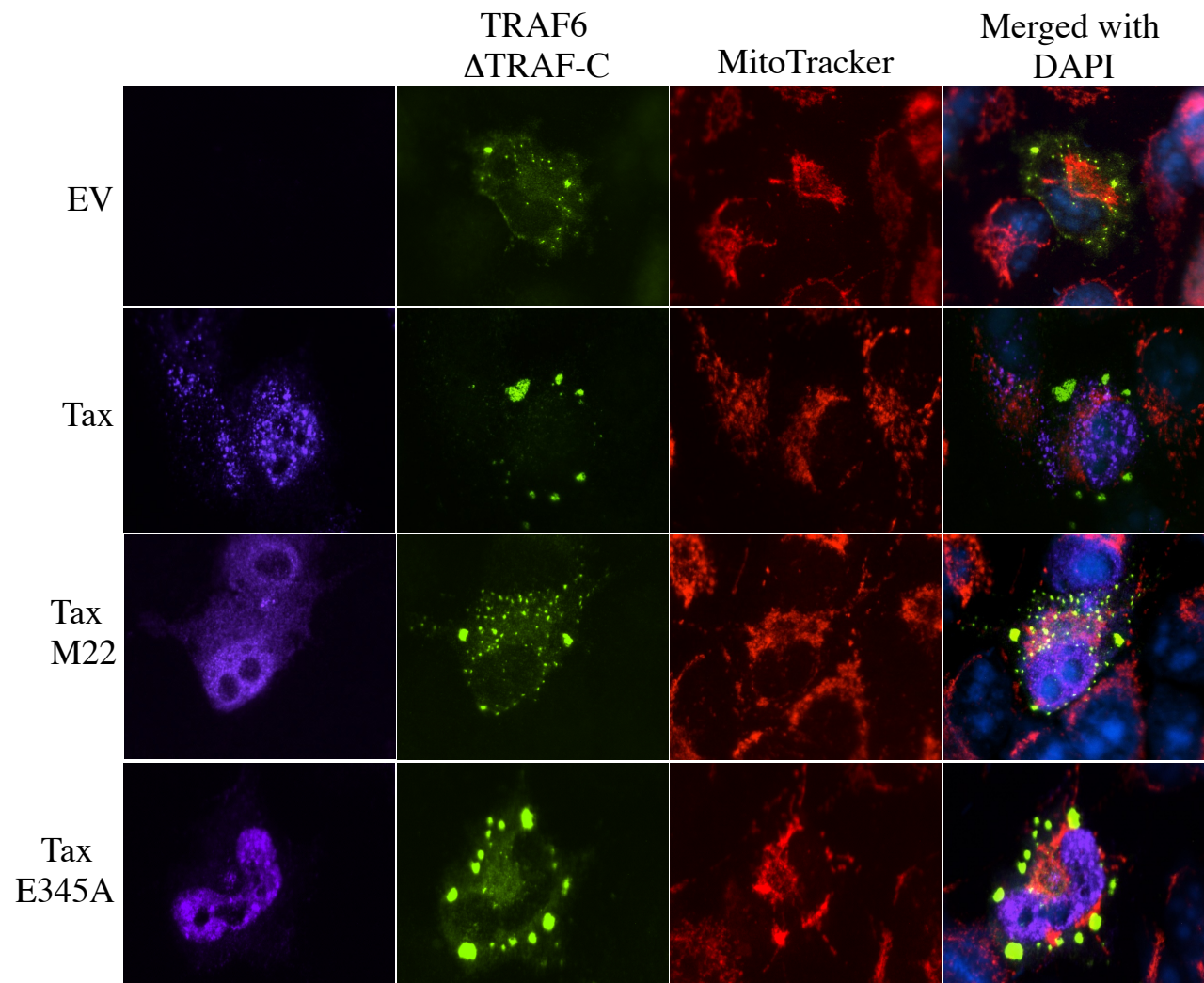

Supplement: Figure S6 — Tax requires the C-terminal TRAF domain of TRAF6 for its mitochondrial localization. Immunofluorescence assay was performed with HeLa cells transfected with Flag-TRAF6ΔTRAF-C together with Tax, TaxM22 or TaxE345A and incubated with MitoTracker Red for 30 min before fixation. Tax and TRAF6 were stained with Alexa Fluor 647 (artificially colored purple) and Alexa Flour 488 (green), respectively. Nuclei were counterstained with DAPI (blue) before mounting coverslips. (PDF) [file ppat.1004458.s006.pdf]

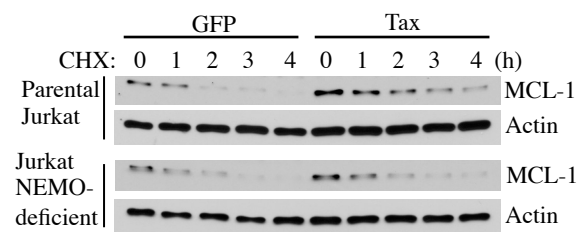

Supplement: Figure S7 — Tax requires NEMO for MCL-1 stabilization. Cycloheximide chase assays were performed by immunoblotting with whole cell lysates derived from wild-type and NEMO-deficient Jurkat cells lentivirally transduced with GFP or Tax at the indicated times after cycloheximide treatment (10 µg/ml). (PDF) [file ppat.1004458.s007.pdf]

**A**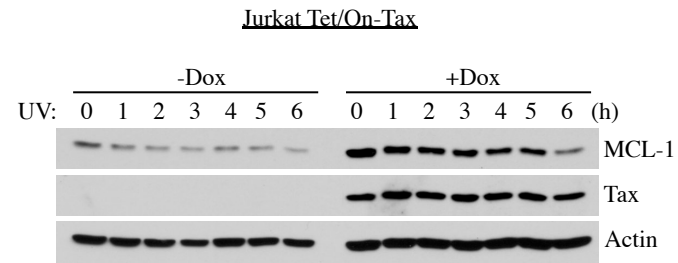**B**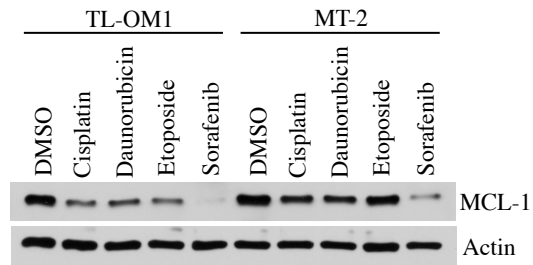

Supplement: Figure S8 — Tax protects MCL-1 from genotoxic stress-induced degradation. (A) Immunoblotting was performed with whole cell lysates derived from Jurkat Tet/On-Tax cells cultured in the presence or absence of doxycycline (Dox, 1 µg/ml) for 48 h followed by UV-irradiation (200 J/m2). (B) Immunoblotting was performed with whole cell lysates derived from TL-OM1 and MT-2 cells treated with cisplatin (25 µM), daunorubicin (5 µM), etoposide (10 µg/ml) and sorafenib (10 µM) for 24 h. (PDF) [file ppat.1004458.s008.pdf]

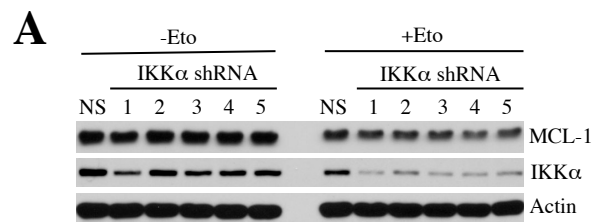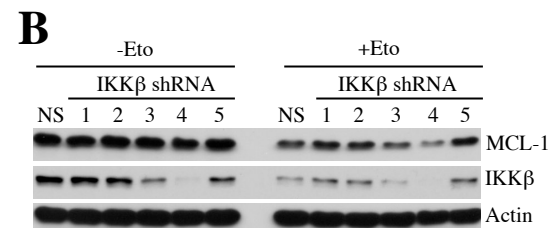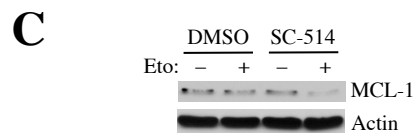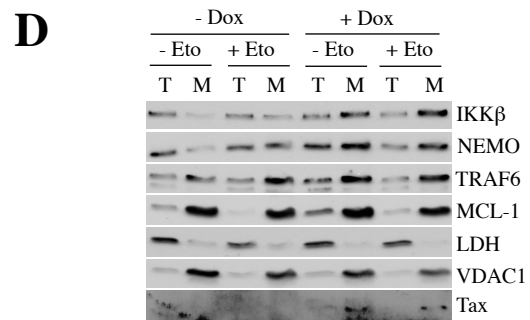

Supplement: Figure S9 — IKK protects MCL-1 from etoposide-induced degradation in HTLV-1 transformed cells. (A and B) Immunoblotting was performed with whole cell lysates derived from MT-2 cells lentivirally transduced with shRNAs specific for IKKα and IKKβ for 3 days and treated with etoposide (10 µg/ml) for 24 h. (C) Immunoblotting was performed with whole cell lysates derived from MT-2 cells pretreated with IKKβ inhibitor SC-514 (20 µM) for 1 h and treated with etoposide for 24 h. (D) Immunoblotting was performed with the indicated fractions derived from Jurkat Tet/On-Tax cells either uninduced or induced with Dox for 48 h. The cells were treated with etoposide (10 µM) as indicated for 6 h before harvesting. A fifty-fold excess of mitochondrial extracts (M) compared to total cell homogenates (T) were loaded for normalization. (PDF) [file ppat.1004458.s009.pdf]

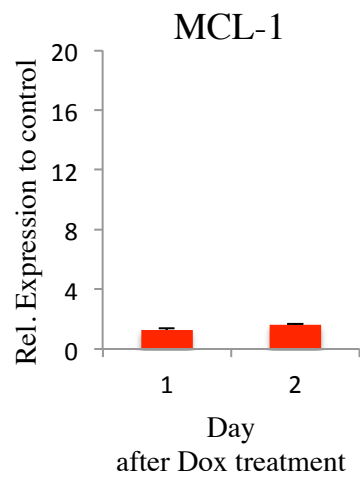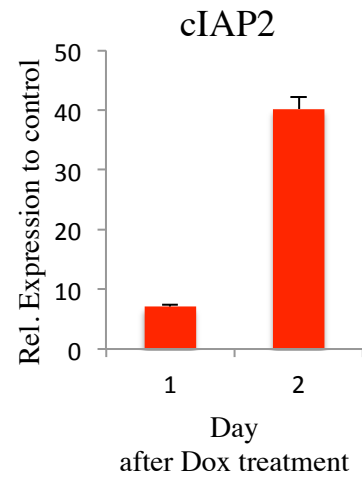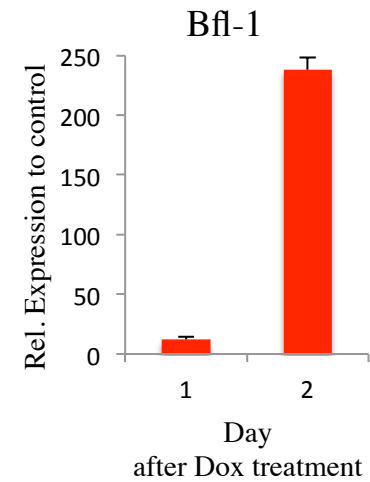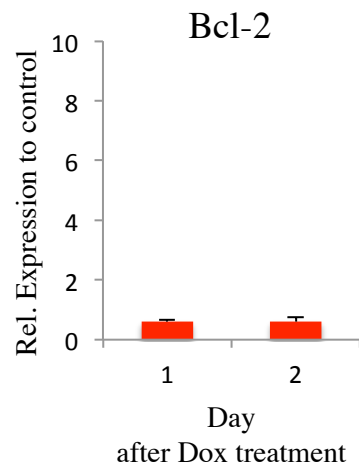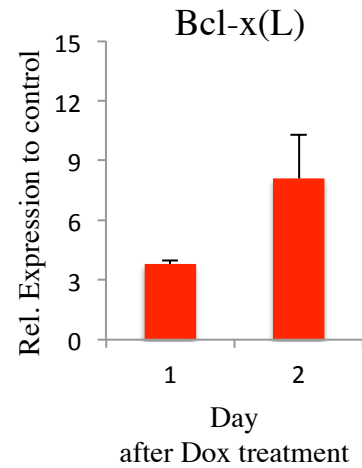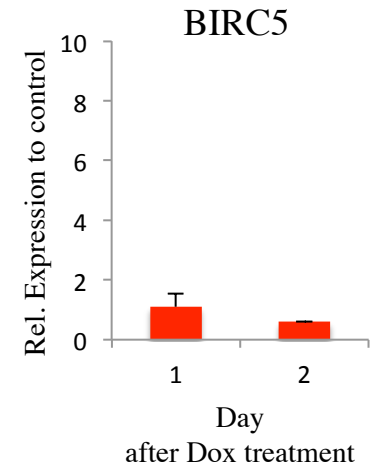

Supplement: Figure S10 — Tax does not transcriptionally regulate MCL-1. qRT-PCR analysis was performed using gene-specific primers with total RNAs isolated from Jurkat Tet/On-Tax cultured with Dox for 0, 1 and 2 days. Graphs depict fold change of mRNA expression relative to cells at 0 days. (PDF) [file ppat.1004458.s010.pdf]

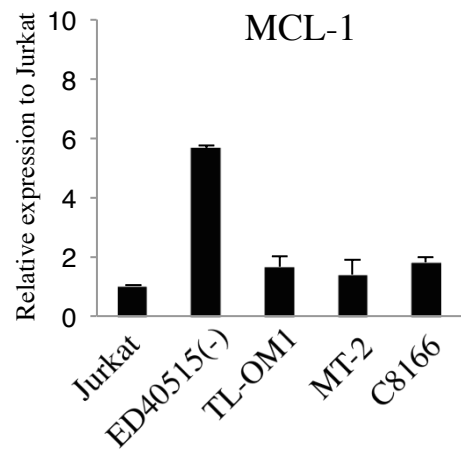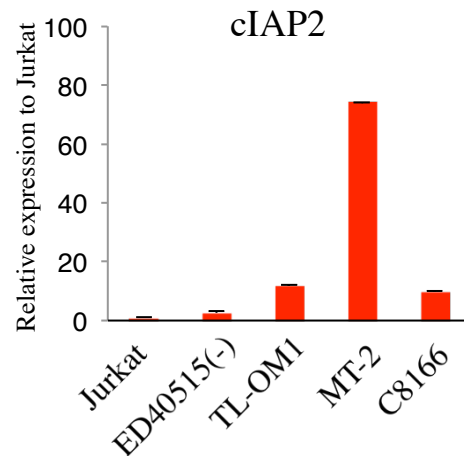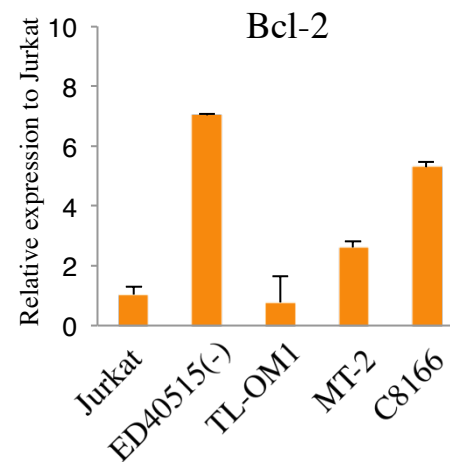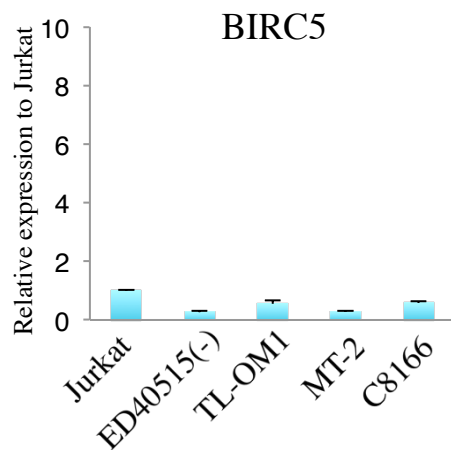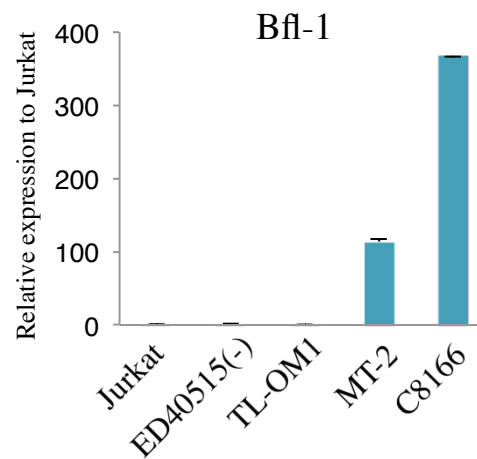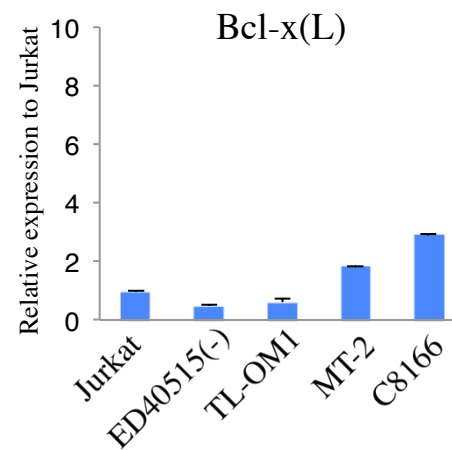

Supplement: Figure S11 — Tax does not regulate MCL-1 mRNA expression in HTLV-1 transformed cell lines. qRT-PCR analysis was performed for the indicated genes with total RNAs isolated from Jurkat, HTLV-1 transformed and ATL cell lines including ED40515(-), TL-OM1, MT-2 and C8166. Graphs depict fold change of mRNA expression relative to Jurkat cells. (PDF) [file ppat.1004458.s011.pdf]

**A**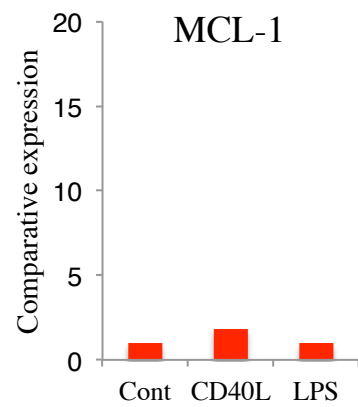**B**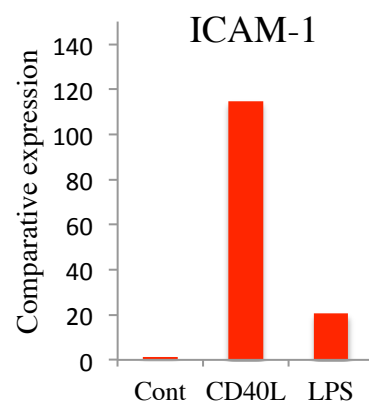**C**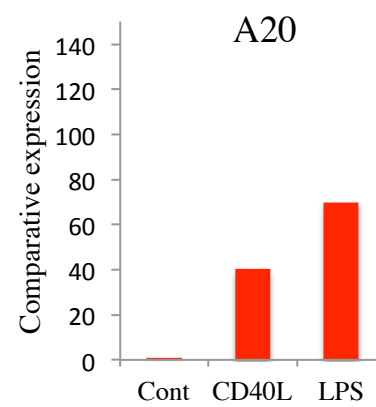

Supplement: Figure S12 — CD40L and LPS do not transcriptionally activate MCL-1 in primary murine B cells. qRT-PCR analysis was performed using gene-specific primers for MCL-1 (A), ICAM-1 (B) and A20 (C) with total RNAs isolated from primary splenic B cells treated with CD40L (100 ng/ml) and LPS (100 ng/ml) for 6 h. Graphs depict fold change of mRNA expression relative to untreated cells. (PDF) [file ppat.1004458.s012.pdf]

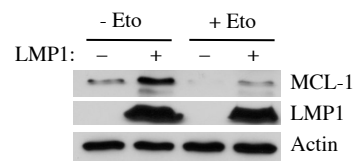

Supplement: Figure S13 — EBV LMP1 protects MCL-1 from etoposide-induced degradation. Immunoblotting was performed with whole cell lysates derived from 293T cells transfected with Flag-MCL-1 together with empty vector or Flag-LMP1 for 24 h and treated with etoposide as indicated for an additional 24 h. (PDF) [file ppat.1004458.s013.pdf]

**A**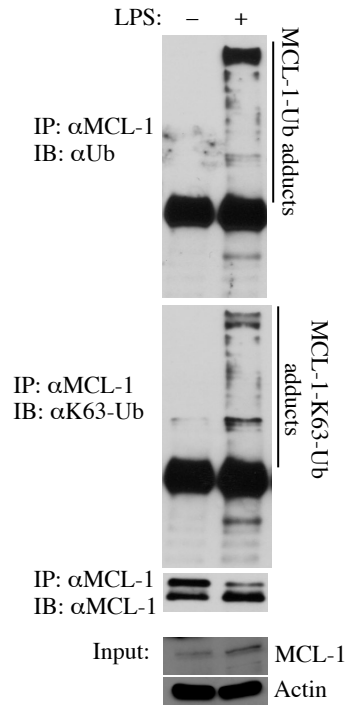**B**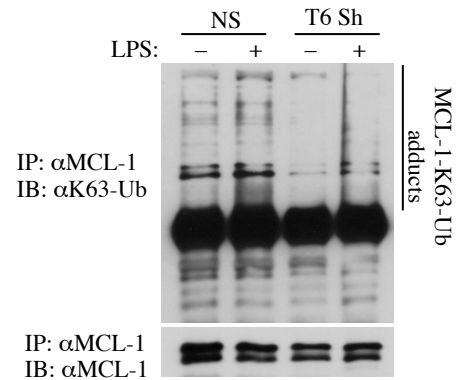

Supplement: Figure S14 — TRAF6 is required for LPS-induced MCL-1 ubiquitination. (A) Ubiquitination assay was performed by IP with α-MCL1 from lysates of RAW 264.7 cells left untreated or treated with LPS (100 ng/ml) for 24 h. Immunoblotting was performed using anti-Ub and K63 Ub antibodies. (B) Ubiquitination assay was performed by IP with α-MCL1 from lysates of RAW 264.7 cells lentivirally transduced with an shRNA targeting mouse TRAF6 for 3 days and treated with LPS (100 ng/ml) for an additional 24 h, and immunoblotting with anti-K63 Ub antibody. (PDF) [file ppat.1004458.s014.pdf]

**A**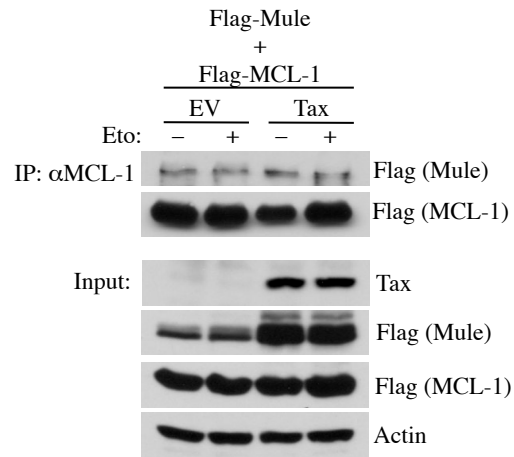**B**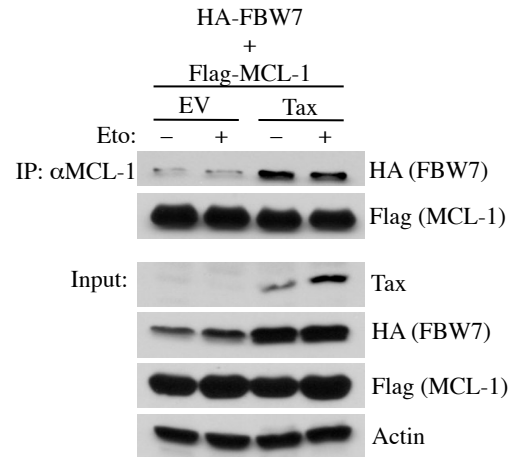**C**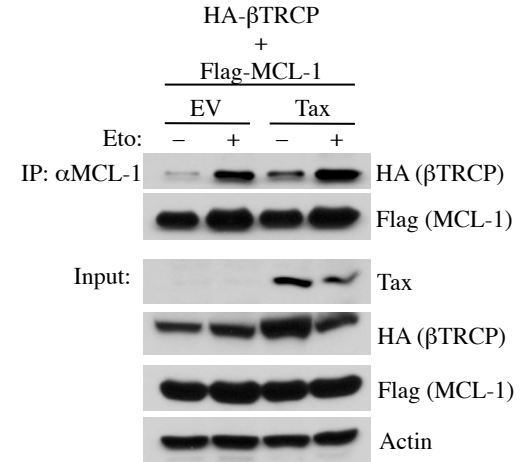

Supplement: Figure S15 — Tax does not impair the interactions between MCL-1 and its degradative E3 Ub ligases. Co-IP was performed with whole cell lysates derived from 293T cells transfected with Flag-MCL-1 together with Flag-MULE (A), HA-FBW7 (B) or HA-βTRCP (C), in the presence or absence of Tax. The cells were left untreated or treated with etoposide (10 µM) for 6 h in the presence of MG-132 (10 µM) before harvesting. (PDF) [file ppat.1004458.s015.pdf]

MCL-1

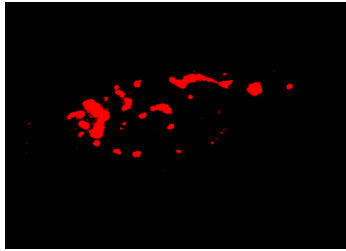

20S proteasome

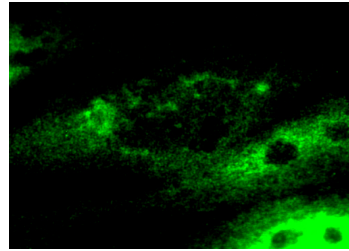

Merged with DAPI

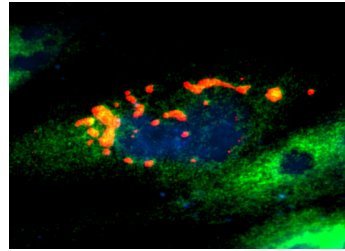

GFP

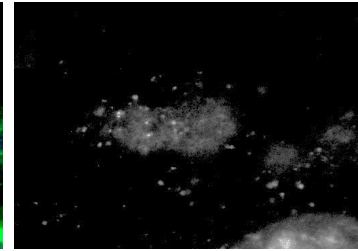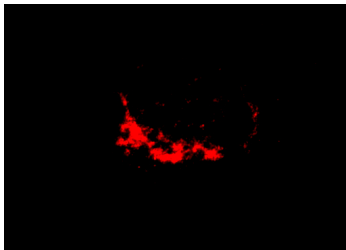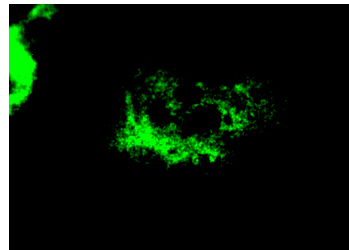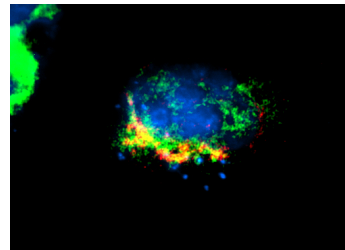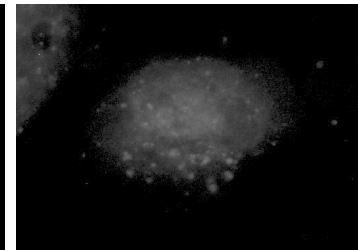

MCL-1

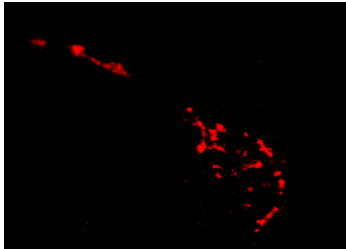

20S proteasome

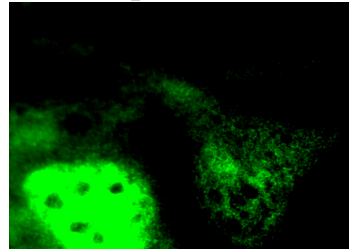

Merged with DAPI

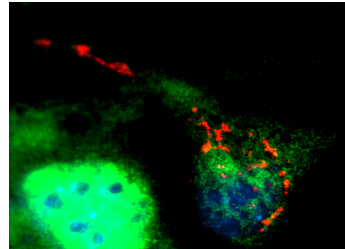

GFP-Tax

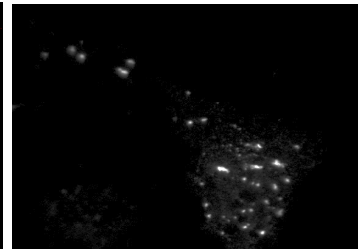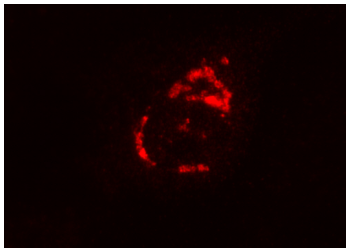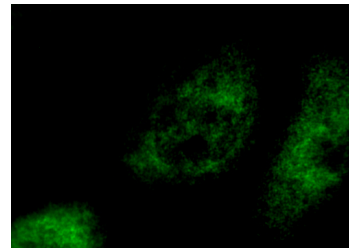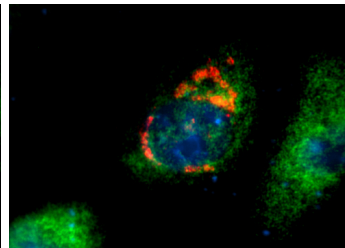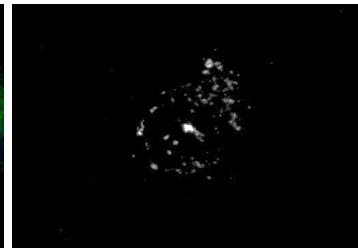

Supplement: Figure S17 — Tax inhibits the co-localization of MCL-1 and the core 20S proteasome (α4 subunit). Immunofluorescence assay was performed with HeLa cells transfected with Flag-MCL-1 together with GFP or GFP-Tax. MCL-1 and 20S were stained with Alexa Fluor 647 (red) and Alexa Fluor 594 (artificially colored green), respectively. The GFP fluorescence signal was changed to gray scale. Nuclei were counterstained with DAPI (blue) before mounting coverslips. (PDF) [file ppat.1004458.s017.pdf]

**A**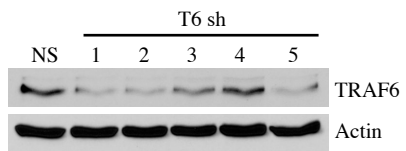**B**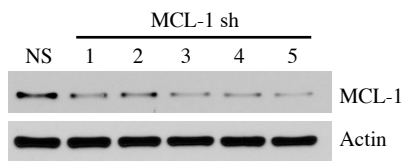**C**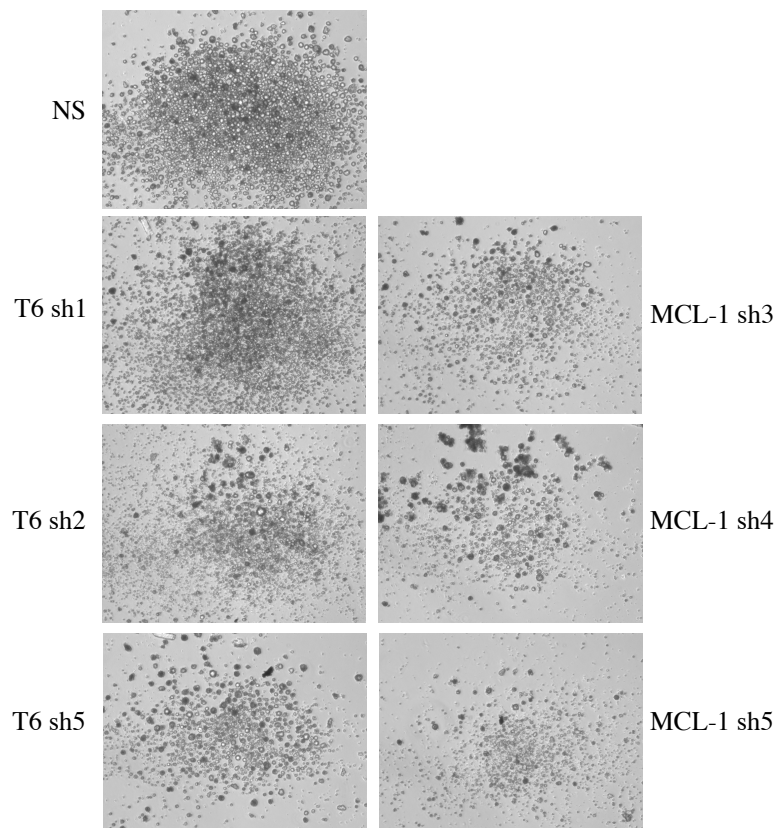

Supplement: Figure S18 — Depletion of TRAF6 and MCL-1 prevent HTLV-1-mediated T-cell immortalization. (A and B) Immunoblotting was performed with whole cell lysates derived from 293T cells transfected for 3 days with TRAF6 (A) or MCL-1 shRNAs (B). (C) Co-culture assay with PBMCs transduced with the indicated shRNAs and irradiated MT-2 cells. Bright field images of the central area of each well were taken 6 weeks after co-culture at 10× magnification. (PDF) [file ppat.1004458.s018.pdf]

**A****HeLa**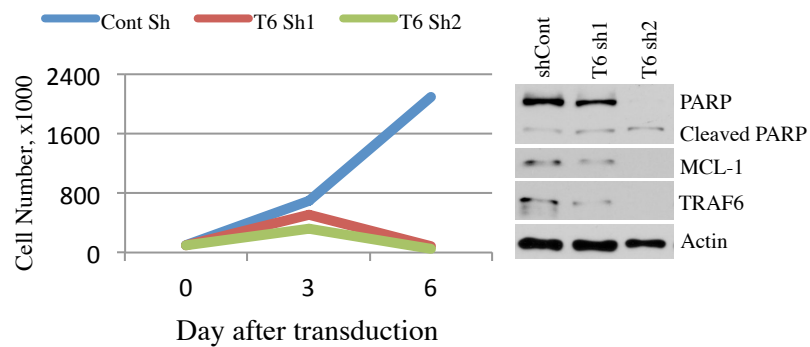**B****MCF-7**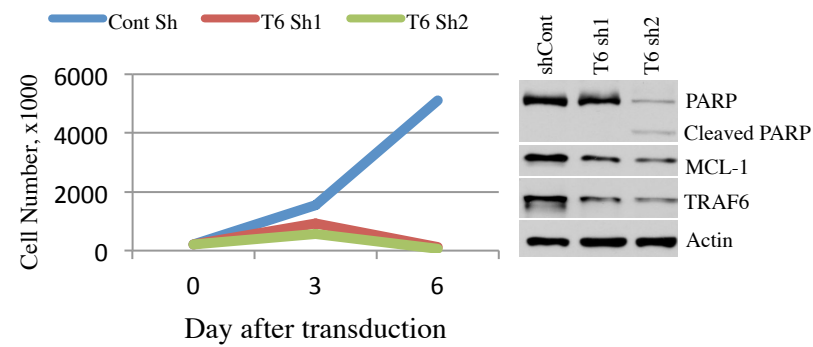**C****DLD-1**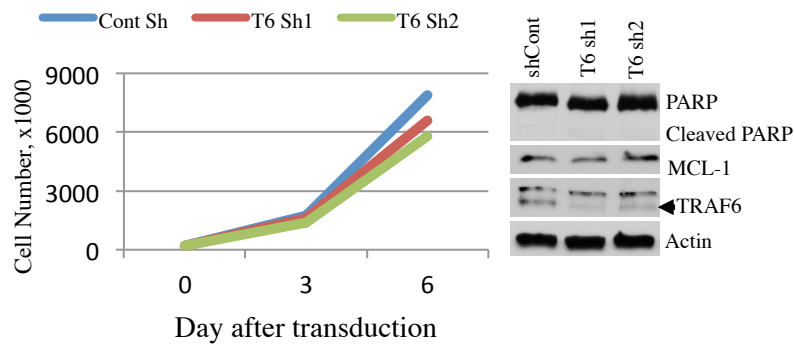**D****293**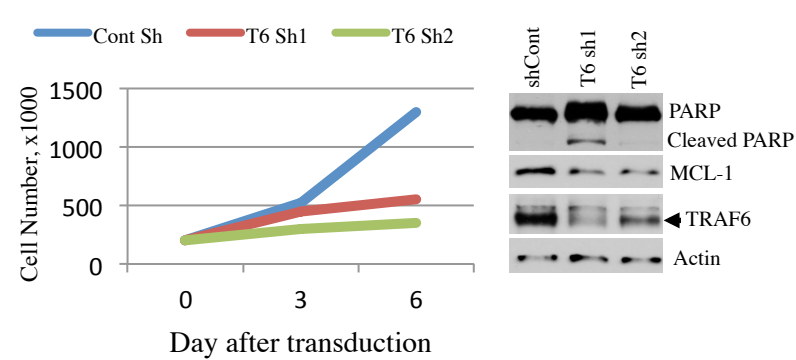

Supplement: Figure S19 — Depletion of TRAF6 attenuates cell growth and promotes apoptotic cell death of cancer cell lines. (A–D) Cell lines including HeLa (A), MCF-7 (B), DLD-1 (C) and 293 (D) were lentivirally transduced with TRAF6 shRNAs, and cell growth was measured by counting the number of viable cells using trypan blue exclusion at the indicated days. Immunoblotting was performed with whole cell lysates of the cells at 3 days after lentiviral transduction using the indicated antibodies. (PDF) [file ppat.1004458.s019.pdf]

**A**

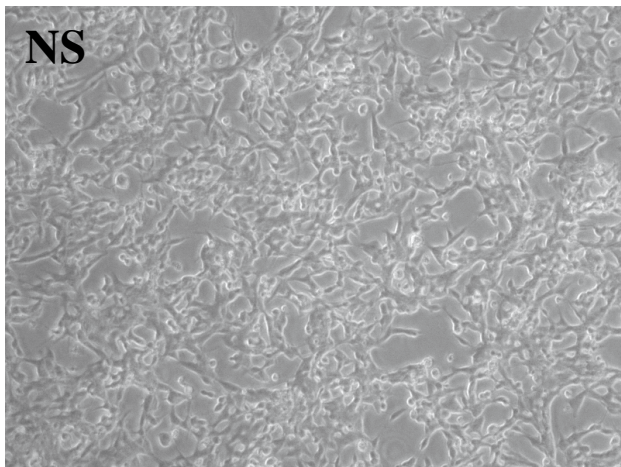

**B**

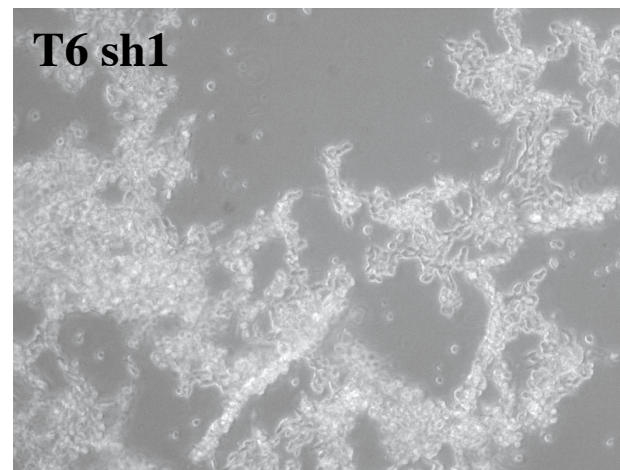

**C**

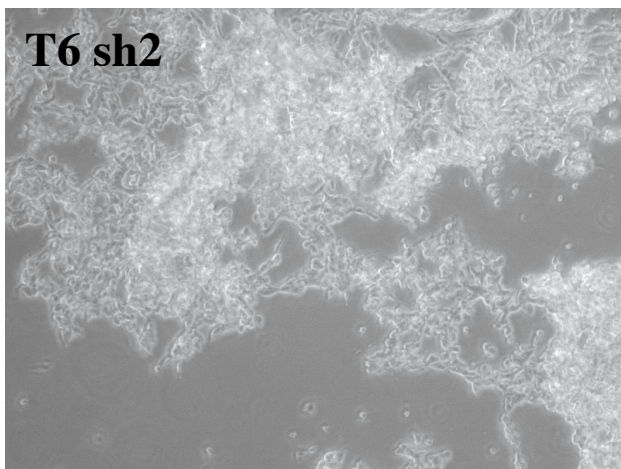

**D**

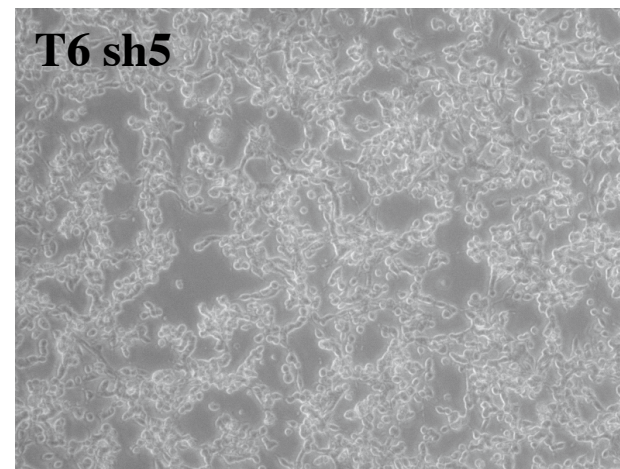

Supplement: Figure S20 — Knockdown of TRAF6 sensitizes cells to sorafenib-induced death. 293 cells were lentivirally transduced with control NS shRNA(A) and TRAF6 shRNAs 1 (B), 2 (C) and 5 (D) and 2 days later treated with sorafenib (1 µM) for 24 h, and bright field microscopic images were taken at 10× magnification. (PDF) [file ppat.1004458.s020.pdf]
