# Supplementary material for: HTLV-1 Tax Stabilizes MCL-1 via TRAF6-Dependent K63-Linked Polyubiquitination to Promote Cell Survival and Transformation
Source: PLoS Pathog. 2014 Oct 23;10(10):e1004458. doi: 10.1371/journal.ppat.1004458 (PMC4207805; doi:10.1371/journal.ppat.1004458)

A

Position of lysine

	5	40	136	194	197	208	234	238	244	276	279	302	308
WT	K	K	K	K	K	K	K	K	K	K	K	K	K
N3-KR	R	R	R	K	K	K	K	K	K	K	K	K	K
N5-KR	R	R	R	R	R	K	K	K	K	K	K	K	K
N6-KR	R	R	R	R	R	R	K	K	K	K	K	K	K
N9-KR	R	R	R	R	R	R	R	R	R	K	K	K	K
N11-KR	R	R	R	R	R	R	R	R	R	R	R	K	K
All-KR	R	R	R	R	R	R	R	R	R	R	R	R	R
C2-KR	K	K	K	K	K	K	K	K	K	K	K	R	R
C4-KR	K	K	K	K	K	K	K	K	K	R	R	R	R
C7-KR	K	K	K	K	K	K	R	R	R	R	R	R	R
C8-KR	K	K	K	K	K	R	R	R	R	R	R	R	R
C10-KR	K	K	K	R	R	R	R	R	R	R	R	R	R

B

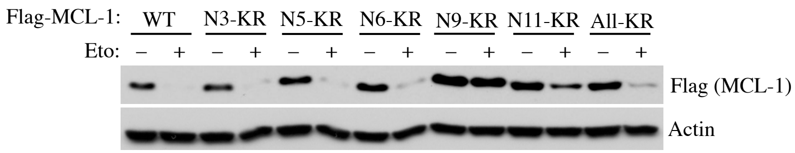

C

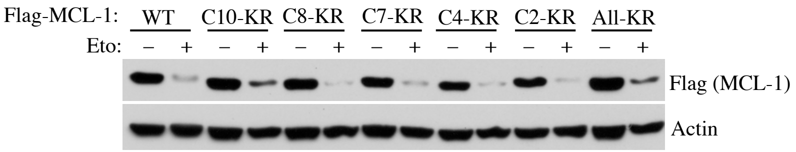

D

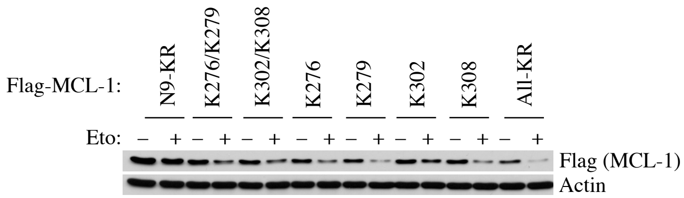

Supplement: Figure S16 — Mapping of MCL-1 lysine residues required for its etoposide-induced degradation. (A) Illustration of MCL-1 lysine to arginine mutants. The substituted arginine (R) residues are highlighted in blue. Immunoblotting was performed with whole cell lysates derived from 293 cells transfected with Flag-MCL-1 mutants in which lysine residues were sequentially replaced with arginine mutated from the N-terminus (B) or C-terminus (C), and left untreated or treated with etoposide (10 µM) for 24 h. (D) Immunoblotting was performed with whole cell lysates of 293 cells transfected with the indicated Flag-MCL-1 plasmids and treated with etoposide for 24 h after transfection. (PDF) [file ppat.1004458.s016.pdf]
