# Supplementary material for: HTLV-1 Tax Stabilizes MCL-1 via TRAF6-Dependent K63-Linked Polyubiquitination to Promote Cell Survival and Transformation
Source: PLoS Pathog. 2014 Oct 23;10(10):e1004458. doi: 10.1371/journal.ppat.1004458 (PMC4207805; doi:10.1371/journal.ppat.1004458)
Supplement: Table S1 — Ubiquitin proteomics results from Jurkat cells inducibly expressing Tax or Tax M22. Summary table of cellular proteins with ubiquitination sites modulated by Tax and/or Tax M22. Green highlight indicates a 2.5-fold or more induction of the indicated ubiquitination site by Tax or Tax M22. Red highlight indicates at least a 2.5-fold decrease of the indicated ubiquitination site by Tax or Tax M22. The protein name is in bold if ubiquitinated in an IKK-dependent manner (by Tax WT but not Tax M22). (PDF) [file ppat.1004458.s021.pdf]

Table S1: Ubiquitin proteomics results

Index	Tax WT	Tax M22	WT:M22	Protein Name	Ub Site
Adaptor/scaffold	-2.3	-2.6	0.3	RAMP	432
	-1.5	3.5	-5.0	FLOT1	126
	-1.5	2.3	-3.8	FLOT1	51
	-1.5	2.6	-4.1	Hrs	107
	-4.5	-1.1	-3.3	OSTF1	162
	2.0	3.4	-1.4	PACSIN3	148
	-1.7	1.6	-3.3	SLA2	202
	2.9	1.8	1.2	**14-3-3 epsilon**	78, 80
Adhesion or extracellular matrix protein	-1.4	-3.9	2.5	CHADL	729, 731
Apoptosis	4.3	3.2	1.1	Bag2	9
Cell cycle regulation	3.1	2.7	0.3	CHMP1A	83
	-1.8	-5.6	3.9	KI-67	254
	-4.0	-1.6	-2.4	NCAPH	126
	-2.5	-1.1	-1.4	SMC2L1	222
	-5.4	-6.7	1.3	NIPA	485
Chaperone	1.0	-4.0	5.0	HSPA4L	53
Chromatin, DNA-binding, DNA repair or DNA replication protein	1.8	3.4	-1.6	CBX3	92
	2.8	2.5	0.3	H1B	161
	2.8	2.6	0.2	H1C	168, 169
	1.3	-1.9	3.3	HMGN2	76, 90
	1.1	-6.6	7.6	MCM7	145
	-1.3	4.2	-5.5	NAP1L1	82
	-1.8	1.5	-3.4	RFC2 iso2	142, 176
	2.8	15.4	-12.6	UBC13	10
	-2.3	-2.6	0.3	ZC3H14	314, 348
Cytoskeletal protein	-2.8	-3.0	0.2	ACTR1A	96
	2.5	2.0	0.5	**Arp3**	42
	2.5	1.8	0.7	**COTL1**	7
	-2.5	-1.5	-1.0	RP1	156
	-1.6	1.9	-3.4	MYO18A iso4	1037, 1038
	-1.8	1.6	-3.5	STMN1	100
	-1.6	-2.9	1.3	TUBA	304, 311, 352, 370
Endoplasmic reticulum or golgi	-2.7	1.4	-4.1	DHRS1	29
Enzyme	-4.5	-3.1	-1.4	DDX3, PL10	262, 264
	2.4	2.5	0.0	FDPS	142
	1.0	-2.6	3.6	FDPS	187
	1.0	-16.8	17.8	FKBP12	35, 36
	-1.3	-3.1	1.9	IDH3B	135
	2.7	15.4	-12.7	NAA50	47
	-1.3	2.5	-3.8	NSUN2	229
	-3.5	-1.3	-2.1	OAS2	404
	1.1	-2.9	4.0	PSAT1	71
	1.7	2.8	-1.1	SUV420H2	442
G protein or regulator	-1.1	3.9	-5.0	DIS3	535
	1.7	4.0	-2.4	G-alpha(13)	214
	-2.5	-1.8	-0.7	TGFBRAP1	370
	-2.8	-1.4	-1.3	VAV1	782
Non-protein kinase	5.1	-1.2	6.3	**PGK1**	275
	2.3	2.9	-0.6	UCK2	78
Lipid binding protein	2.7	9.3	-6.6	pleckstrin	101
Motor or contractile protein	-2.7	-1.5	-1.2	MYO1G	507, 603
Phosphatase	-2.6	-1.6	-1.1	LANP-L	116
	-1.0	-2.5	1.4	PPP2R1	188, 200
	-2.6	1.4	-4.0	SHP-1 iso5	271
	-3.5	-1.9	-1.6	SHP-1 iso5	360
	-1.6	1.6	-3.2	RNF213	1569
Protease	-1.2	-3.4	2.2	APEH	681
	1.4	-3.1	4.5	PSMA1	30
	-2.6	-1.5	-1.1	PSMD12	52
	4.0	-1.2	5.2	**TNFAIP3**	81, 424, 520, 541, 643, 676, 690, 722, 786

Table S1: Ubiquitin proteomics results (*continued*)

Index	Tax WT	Tax M22	WT:M22	Protein Name	Ub Site
Protein kinases	-2.7	-1.2	-1.5	PITSLRE B	111, 455, 467
	-2.7	-2.9	0.1	CDK5	268
	3.0	2.9	0.0	eEF2K	341, 347
	1.3	3.2	-1.9	TLK2	361
	-1.0	-3.8	2.8	ITK	129, 497
	-2.8	-1.4	-1.3	Lck	401, 405
	-2.6	-1.2	-1.4	ZAP70	132, 542
	-2.2	-2.6	0.5	ZAP70	603, 613
	1.4	3.4	-2.0	Mer	856
	-3.0	-2.4	-0.5	TRIM28	266
	-3.0	1.0	-4.0	AMPKG2	9
RNA processing	1.7	-2.3	4.0	DZIP3	248, 768, 936, 1087
	-1.9	-2.5	0.6	eIF4A1, DDX48	190, 284, 285, 289
	-2.6	-1.5	-1.1	hnRNP A0	159, 172
	2.5	1.5	0.9	**PRPF8**	727
	-1.6	-3.7	2.1	RALY	13, 143, 159
Receptor, channel, transporter or cell surface protein	-1.2	-2.7	1.5	CD3Z	104, 136
	1.1	-5.6	6.7	CD3Z	116
	3.2	2.8	0.3	DPAGT1	48
	-1.5	-2.5	1.1	HCST	83
	-1.7	-5.6	3.9	HCST	84
	2.9	1.0	1.8	**HLAA iso3**	340, 364
	-1.6	-4.2	2.6	hnRNP M	349, 388
	4.7	3.0	1.7	KCTD10	25, 32
	5.7	5.0	0.6	LAPTM5	233
	4.5	1.6	2.9	**MCL1**	40
	3.2	2.1	1.1	**MT**	31, 32
	4.7	2.1	2.7	**MT1E**	51
	4.7	2.1	2.7	**MT2A**	51
	4.0	-1.0	5.0	**NSDHL**	96
	2.9	3.0	0.0	SCAMP3 iso2	75, 76, 101, 102
	7.8	1.6	6.2	**SEMA4A**	727
	-1.1	3.9	-5.0	SLC35F5	191
	-1.2	2.5	-3.7	SNX13	635
	2.6	1.2	1.4	**STX4**	58
	-2.6	-8.4	5.8	TMEFF1	365
	5.6	2.0	3.6	**TPR**	1690
	1.0	-4.4	5.4	tubby	352
Transcriptional regulator	-2.9	1.1	-4.0	AIP	210
	-2.6	-1.3	-1.2	Tel	393
	1.1	-2.5	3.5	hnRNP P2	451
	-1.8	-2.5	0.7	hnRNP U iso2	497, 516
	-2.8	-1.1	-1.7	IFI16	64
	2.1	-1.6	3.7	Aiolos	245
	-1.7	1.6	-3.2	MGMT	125
	-2.2	1.9	-4.1	Myc	148
	3.0	3.7	-0.6	PARP14	1589
	1.2	-3.6	4.8	PATZ1 iso4	272
	-2.6	-4.0	1.4	PSF	559
	3.0	2.5	0.6	PC4	53
	-3.5	-2.7	-0.8	TAFII31 iso2	21
	-1.0	-3.7	2.6	UBE2L3 iso2	9
	5.6	4.1	1.4	UHRF1	303
	3.3	-2.2	5.5	**BRF1**	72
	-2.1	-5.5	3.4	ZNF451	858
Translation	-1.3	1.9	-3.3	CAPG	137
	-1.4	2.4	-3.8	CAPG	138
	-9.0	-11.7	2.7	eEF1G	17
	4.8	1.8	3.0	**eIF3S3**	24
	2.0	2.6	-0.5	eIF3S3	5
	-1.1	2.8	-3.9	IMP-1	190
	3.3	13.2	-9.9	RPL11	67
	-1.6	-2.7	1.2	RPL15	153
	1.7	-2.9	4.5	RPL29	156
	3.7	6.5	-2.9	RPS27A, UBA52	27, 103, 179, 255, 331, 407, 483, 559, 635

Table S1: Ubiquitin proteomics results (*continued*)

Index	Tax WT	Tax M22	WT:M22	Protein Name	Ub Site
Ubiquitin conjugating system	4.5	-1.0	5.5	**cIAP2**	218, 231
	2.2	5.5	-3.3	HUWE1	2695
	-2.2	-4.9	2.7	HUWE1	3370
	2.4	-1.0	3.4	RNF168	528
	1.3	10.7	-9.4	RNF213	520
	1.5	-2.1	3.6	XIAP	31
Unknown function	-2.6	-1.9	-0.7	ANKFY1	1088
	-2.9	-1.6	-1.3	ARMC6	125
	2.8	-5.5	8.3	**FBXL20**	10
	-3.8	1.3	-5.1	GIMAP7	158
	1.2	-2.4	3.7	H3	57
	2.4	10.0	-7.7	KLHL26	447
	-2.0	-2.8	0.9	MGC16703	78, 164
	-2.6	-2.5	-0.1	PGBD2	459
	2.5	-1.0	3.5	**PPDPF**	99
	3.5	1.2	2.4	**RFTN1 iso2**	132, 147
	2.0	3.3	-1.3	SR-A1	55
	2.5	1.8	0.7	**THYN1**	47
	-3.2	-1.6	-1.6	TTL12	100
	-3.1	-1.7	-1.4	TTL12	342
Vesicle protein	5.5	3.7	1.8	VPS45A	557
