# Supplementary material for: HTLV-1 Tax Stabilizes MCL-1 via TRAF6-Dependent K63-Linked Polyubiquitination to Promote Cell Survival and Transformation
Source: PLoS Pathog. 2014 Oct 23;10(10):e1004458. doi: 10.1371/journal.ppat.1004458 (PMC4207805; doi:10.1371/journal.ppat.1004458)
Supplement: Table S2 — Oligonucleotide sequences for shRNAs. (PDF) [file ppat.1004458.s022.pdf]

Table S2: Oligonucleotide sequences for shRNAs

Name	Target sequences
TRAF6 sh1	5’- GCCACGGGAAATATGTAATAT -3’
TRAF6 sh2	5’- CGGAATTTCCAGGAAACTATT -3’
TRAF6 sh3	5’- CGAAGAGATAATGGATGCCAA -3’
TRAF6 sh4	5’- CCCATCTGCTTGATGGCATTA -3’
TRAF6 sh5	5’- CCTGGATTCTACACTGGCAAA -3’
Murine TRAF6 sh	5’- CCCAGGCTGTTCATAATGTTA -3’
MCL-1 sh1	5’- GCCTTCCTCACCAATGTTCCC -3’
MCL-1 sh2	5’- GTTCCCTACAAGCGAATAGAA -3’
MCL-1 sh3	5’- GCCTACCACCCCTCATTTCTA -3’
MCL-1 sh4	5’- GAGGCAGATGACAATGACCAT -3’
MCL-1 sh5	5’- GCTTAGAGCCTCCCAGTGAAA -3’
Tax sh1	5’- GCCTTCCTCACCAATGTTCCC -3’
Tax sh2	5’- GTTCCCTACAAGCGAATAGAA -3’
Tax sh3	5’- GCCTACCACCCCTCATTTCTA -3’
Tax sh4	5’- GAGGCAGATGACAATGACCAT -3’
Tax sh5	5’- GCTTAGAGCCTCCCAGTGAAA -3’
IKKα sh1	5’- TGAATGTATTGCTGGATATAG -3’
IKKα sh2	5’- TAGGGTCTGGGATTCGATATT -3’
IKKα sh3	5’- ACAGCGTGCCATTGATCTATA -3’
IKKα sh4	5’- TGGCCATTTAAGCACTATTAT -3’
IKKα sh5	5’- GGTTAATGTAGTATGGTATAT -3’
IKKβ sh1	5’- CAAGGAGAACAGAGGTTAATA -3’
IKKβ sh2	5’- GTATTTCAGACGGCAAGTTAA -3’
IKKβ sh3	5’- ACAGCGAGCAAACCGAGTTTG -3’

IKKβ sh4	5’- CATGAATGCCTCTCGACTTAG -3’
IKKβ sh5	5’- GGCAGTCTTTGCACATCATTC -3’
