# Supplementary material for: HTLV-1 Tax Stabilizes MCL-1 via TRAF6-Dependent K63-Linked Polyubiquitination to Promote Cell Survival and Transformation
Source: PLoS Pathog. 2014 Oct 23;10(10):e1004458. doi: 10.1371/journal.ppat.1004458 (PMC4207805; doi:10.1371/journal.ppat.1004458)
Supplement: Table S3 — Primer sequences for qRT-PCR. (PDF) [file ppat.1004458.s023.pdf]

Table S3: Primer sequences for qRT-PCR

Name	Forward	Reverse
MCL-1	5'- TGCTTCGGAAACTGGACATCA -3'	5'- TAGCCACAAAGGCACCAAAAG -3'
cIAP2	5'- TTTCCGTGGCTCTTATTCAAACT -3'	5'- GCACAGTGGTAGGAACTTCTCAT -3'
Bfl-1	5'- AGTGCTACAAAATGTTGCGTTC -3'	5'- GGCAATTTGCTGTCGTAGAAGTT -3'
Bcl-2	5'- GGTGGGGTCATGTGTGTGG -3'	5'- CGGTTCAGGTACTCAGTCATCC -3'
Bcl-XL	5'- GAGCTGGTGGTTGACTTTCTC -3'	5'- TCCATCTCCGATTCAGTCCCT -3'
BIRC5	5'- AGGACCACCGCATCTCTACAT -3'	5'- AAGTCTGGCTCGTTCTCAGTG -3'
18S rRNA	5'- GTAACCCGTTGAACCCCATT -3'	5'- CCATCCAATCGGTAGTAGCG -3'
Murine MCL-1	5'- AAAGGCGGCTGCATAAGTC -3'	5'- TGGCGGTATAGGTCGTCCTC -3'
Murine ICAM-1	5'- GTGATGCTCAGGTATCCATCCA -3'	5'- CACAGTTCTCAAAGCACAGCG -3'
Murine A20	5'- GAACAGCGATCAGGCCAGG -3'	5'- GGACAGTTGGGTGTCTCACATT -3'
Murine 18S rRNA	5'- AGGGGAGAGCGGGTAAGAGA -3'	5'- GGACAGGACTAGGCGGAACA -3'
